# Evidence for Environmental Noise Effects on Health for the United Kingdom Policy Context: A Systematic Review of the Effects of Environmental Noise on Mental Health, Wellbeing, Quality of Life, Cancer, Dementia, Birth, Reproductive Outcomes, and Cognition

**DOI:** 10.3390/ijerph17020393

**Published:** 2020-01-07

**Authors:** Charlotte Clark, Clare Crumpler, Hilary Notley

**Affiliations:** 1 Acoustics, Ove Arup & Partners, 13 Fitzroy Street, London W1T 4BQ, UK; 2 UK Department for the Environment, Food and Rural Affairs (Defra), Ground Floor, Seacole Building, 2 Marsham Street, London SW1P 4DF, UK; noise@defra.gov.uk

**Keywords:** road traffic noise, aircraft noise, railway noise, quality of life, wellbeing, mental health, cancer, birth outcomes, dementia, children’s learning

## Abstract

This systematic review commissioned by the UK Department for the Environment, Food and Rural Affairs (Defra), considers how the evidence base for noise effects on health has changed following the recent reviews undertaken for the WHO Environmental Noise Guidelines. This systematic review assesses the quality of the evidence for environmental noise effects on mental health, wellbeing, and quality of life; birth and reproductive outcomes; and cognition for papers published since the WHO reviews (mid-2015 to March 2019), as well as for cancer and dementia (January 2014 to March 2019). Using the GRADE methodology (Grading of Recommendations Assessment, Development and Evaluation) most evidence was rated as low quality as opposed to very low quality in the previous reviews. There is now low-quality evidence for a harmful effect of road traffic noise on medication use and interview measures of depression and anxiety and low quality evidence for a harmful effect of road traffic noise, aircraft noise, and railway noise on some cancer outcomes. Many other conclusions from the WHO evidence reviews remain unchanged. The conclusions remain limited by the low number of studies for many outcomes. The quantification of health effects for other noise sources including wind turbine, neighbour, industrial, and combined noise remains a research priority.

## 1. Introduction

The publication of the World Health Organization’s (WHO) Environmental Noise Guidelines for the European Region was accompanied by a number of systematic evidence reviews detailing the strength of the evidence for the effects of environmental noise on annoyance [1], sleep [2], cardiovascular health [3]; birth and reproductive outcomes [4]; cognition [5]; and mental health, wellbeing, and quality of life [6], amongst others. These influential systematic reviews of the strength of the evidence informed the setting of the WHO guidelines.

In the United Kingdom (UK), the current guidance for economically valuing the impacts associated with environmental noise is published by Defra (the Department for the Environment, Food, and Rural Affairs) on behalf of the Interdepartmental Group on Costs and Benefits (Noise Subject Group) (IGCB(N)), with the current guidance relying on evidence for noise and health effects published up to 2014 [7,8]. This guidance has subsequently been used to inform the government’s Transport Appraisal Guidance for noise, [9] and Her Majesty’s Treasury Green Book on appraisal and evaluation in central government [10], both of which monetise the effects of noise on health. The existing guidance covers the effects of aircraft noise, road traffic noise, or railway noise on acute myocardial infarction; amenity (annoyance); stroke; vascular dementia and sleep disturbance.

Following publication of the WHO Environmental Noise Guidelines for the European Region [11], the IGCB (N) was convened to consider any necessary updates to relevant government guidance. This led Defra to commission additional systematic reviews, to consider how the evidence base has changed since the WHO systematic reviews were published in 2017 and 2018. These reviews will inform the convened IGCB (N) on whether additional evidence should be considered beyond that considered in the WHO systematic reviews.

This paper reports the methodology and findings of systematic reviews that assess the quality of the evidence for the effect of environmental noise (road traffic noise, aircraft noise, railway noise, wind-turbine noise, and other noise sources) on mental health, wellbeing and quality of life; birth and reproductive outcomes; dementia and other neurodegenerative conditions; and cognition for papers published since the WHO reviews (from mid-2015 up to March 2019). The previous review undertaken for the WHO for mental health, wellbeing, and quality of life concluded that most evidence was very low quality, with effects only being observed for some noise sources and outcomes [6]. The previous review undertaken for the WHO found that there was moderate quality evidence for an effect of road traffic and railway noise on emotional and conduct disorders and hyperactivity in children, as well as very low quality evidence for an effect of aircraft noise on medication use and interview measures of depression and anxiety. For cognition, the previous review undertaken for the WHO found the quality of the evidence across studies ranged from being of moderate quality for an effect for some outcomes—e.g., aircraft noise effects on reading comprehension and on long-term memory—to no effect for other outcomes such as attention and executive function and for some noise sources, such as road traffic noise and railway noise [5]. For reproductive outcomes, the previous review undertaken for the WHO found very low quality evidence for associations between aircraft noise and preterm birth, low birth weight and congenital anomalies, and low quality evidence for an association between road traffic noise and low birth weight, preterm birth and being small for gestational age [4]. All three reviews undertaken for the WHO identified the need for further studies for these outcomes, and a need for longitudinal studies.

The scope also included additional reviews on health outcomes not considered by the WHO. The evidence for cancer was assessed as several papers on environmental noise and cancer have emerged since the cut-off date of the last Defra review (2014) (e.g., [12,13]). Defra also requested a review of the evidence for dementia and other neurodegenerative outcomes, as the existing guidance includes dementia based on the evidence at that time showing a link between firstly, environmental noise and hypertension and secondly, between hypertension and vascular dementia. Several papers examining the direct association between environmental noise exposure and vascular dementia have been published since 2014 (e.g., [14,15]).

## 2. Materials and Methods

### 2.1. Scope of the Review

The methodology mirrors that used in the recent systematic reviews carried out to inform the World Health Organization’s (WHO) Environmental Noise Guidelines for the European Region [11], where possible [4,5,6].

The review sought to identify original research papers of quantitative design, on the effect of environmental noise on mental health, wellbeing, and quality of life; cancer; birth and reproductive outcomes; dementia and other neurodegenerative diseases; and cognition. Papers were sought that used epidemiological methods, including survey, case-control studies and cohort studies. Following the approach taken for the WHO reviews, experimental studies were excluded as the review focused on the long-term effects of chronic environmental noise exposure on these outcomes, as opposed to effects of acute environmental noise exposure in the laboratory. This has particular relevance to the search for cognition where experimental methods are commonly used.

The systematic reviews for each of the health outcomes were carried out for the time-periods shown in Table 1 based on the publication end-dates of the existing reviews (mental health, cognition, birth, and reproductive outcomes) or for the last four years (since 2014—the date of the last ICGB (N) review) where existing reviews were not available (dementia, cancer).

Search terms covering different sources of environmental noise (aircraft, road traffic, railway, wind-turbine, building services noise, industrial noise, etc. (see Appendix A.1 for details), different study designs (cross-sectional, longitudinal), and different outcomes were included in database searches of Medline/PubMed and ScienceDirect (see Appendix A.1 for the complete list of search terms included). The search terms used were based upon those used in the previous WHO systematic reviews on these health topics, where available [4,5,6]. For dementia and other neurodegenerative diseases and cancer, we set up search terms using key words (see Appendix A.1).

The searches were undertaken for the following environmental noise sources (covering a range of noise metrics) including road; rail, aircraft, windfarms/wind turbines, industrial, noise from building services equipment including ground and air source heat pumps; neighbour noise and neighbourhood noise. Papers examining other types of noise exposure, such as occupational noise or hospital noise were excluded, as per the approach taken in the reviews undertaken for the WHO. Papers that did not characterise noise using established methods, i.e., by measurement or modelling were excluded (e.g., studies that use distance to roads as a proxy for noise exposure).

### 2.2. Search Strategy

Quantitative papers in English were sought but due to time constraints, conference proceedings were not additionally systematically searched. The reference lists of identified papers were checked for further relevant citations. Grey literature, already known to the authors was also included in the review. Papers from Internoise 2019 (June 2019) and ICA2019 (September 2019) were added to the review after the searches had been completed, where relevant.

### 2.3. Data Screening and Review Process

Papers were reviewed in two stages. First, all the titles and abstracts of the identified papers were reviewed by two reviewers (CC1, CC2) separately to assess eligibility for inclusion in the review. Second, the full text of eligible papers was retrieved and two reviewers (CC1, CC2) read the paper and re-assessed eligibility for inclusion. At both stages, where there was disagreement between the reviewers’ discussion was held until consensus reached.

### 2.4. Data Extraction

The eligibility criteria matched those used by the reviews undertaken for the WHO, covering the aspects listed below. Papers which failed to meet any one of the (PECO) inclusion criteria or which met one of the exclusion criteria were excluded from the review.

- Population: the inclusion criteria were studies of the general population or specific sub-groups of the population in settings (home, public venues, schools).
- Exposure: the inclusion criteria were exposure to high levels of environmental noise from the sources specified above. Included studies either measure or calculate noise exposure levels expressed in decibel values at an appropriate location for the study participants (e.g., home, school). Where calculated levels were available for transportation noise, they reflected the use of roads, railways lines and flight routes. Exclusion criteria included studies using distance to source as a proxy for noise exposure and studies using subjective ratings of noise exposure (including noise annoyance) as a proxy for noise exposure.
- Comparator: the inclusion criteria were that the study has a comparator group with no noise exposure or a lower level of noise exposure.
- Assessment of outcome: the inclusion criteria were that the outcome data came from medical records or interviews or cognitive testing using a known scale or validated assessment method or that the outcome was self-reported from a questionnaire.

Papers were identified for inclusion in the systematic review regardless of the study findings, i.e., all papers, regardless of whether they found a significant positive or negative association between environmental noise and the health outcome or whether they found no effect were considered in the review.

Each paper was subsequently assessed for the following types of bias:

1. Noise exposure assessment leading to information bias: evaluating whether the paper used established noise metrics in dB; the timeframe of noise measurements, if applicable; and quality of noise modelling, if applicable.
2. Bias due to confounding: evaluating whether the study used matching or adjustment in the analysis for potential confounding factors, such as socioeconomic status, which can influence both noise exposure and the health outcome.
3. Bias due to selection of participants: whether participants are randomly sampled from a known population and whether the response rate was higher than 60%. Consideration of bias associated with drop out for longitudinal studies.
4. Outcome assessment leading to information bias I: whether the assessment of the outcome is objectively measured using a known scale or validated measure.
5. Outcome assessment leading to information bias II: whether the assessment is blinded for exposure information in cohort.

Ratings for each type of bias were low bias, unclear, or high bias. Bias was considered present for each aspect noted above, if this information was omitted from the paper. Many studies fail to report a response rate for their study, which results in the study being assigned a rating as ‘unclear’ for bias due to the selection of participants. Table 2, Table 3, Table 4, Table 5 and Table 6 summarise the bias ratings for each individual study included in the review.

### 2.5. Evaluating the Quality of the Evidence

The previous reviews of these cognitive and health outcomes have struggled to identify enough papers to warrant the use of meta-analysis and have also identified problems of using meta-analysis when the outcome measures vary greatly across the studies—e.g., for cognition, and mental health and wellbeing [5,6]. The scope of the current reviews was to undertake a narrative review of the evidence, rather than a meta-analysis. The narrative review considers the evidence for each noise source separately in relation to the range of outcomes identified for a specific health or cognitive outcome. A narrative approach enables comparison with the evidence from the recent systematic reviews carried out for the WHO for these outcomes.

The GRADE methodology [16], recommended by the Cochrane Collaboration [17] and by the WHO Handbook for Guideline Development [18], and adapted for use in the WHO Environmental Noise Guidelines for the European Region [11] was used to interpret the body of evidence for each noise source and outcome. The GRADE methodology ranks the quality of evidence as high, moderate, low, or very low. The GRADE methodology is not used to rate individual studies but is used to rate the overall quality of evidence available for a specific environmental noise source and health outcome—that is all the studies available, regardless of whether they find a significant statistical effect of environmental noise on a specific health outcome or not. The GRADE assessment was undertaken individually for each environmental noise and health outcome, even if only one study was available.

The adapted GRADE methodology assigns the highest quality of evidence available to longitudinal or intervention studies and where only cross-sectional studies are available the evidence is initially judged as being of low quality. The GRADE methodology allows for these initial evidence ratings to be further upgraded or downgraded according to specific criteria. Upgrades can be made based on the availability of evidence for an exposure–response function (ERF) between noise and the health outcome; the magnitude of the relative risk being >2; or there being evidence for an effect in spite of confounding working towards the null. However, in practice, upgrades for confounding are very rarely made and no upgrading of the evidence for this factor has taken place in this review. Downgrades can be made based on most of the studies being of low quality (study design); inconsistent findings between studies (inconsistency); studies not comparing the same outcomes (indirectness); effect estimate confidence interval containing 25% harm or benefit (precision); or publication bias, as assessed by a funnel plot. Unfortunately, it is not possible to assess precision and publication bias when undertaking a narrative review as these assessments require the statistical outcome from meta-analyses.

The GRADE methodology is accompanied by a statement as to whether the body of evidence suggests there is an effect of environmental noise on the health outcome or if there is no effect. Drawing on the approach of previous ICGB(N) reports, where individual studies carried weight in terms of establishing whether there is or is not an effect of noise on a health outcome, in this review, taking a precautionary approach an ‘effect’ has been identified even where there is only one study available within the body of evidence. This approach may result in an over-statement of whether there is an effect or not as it ignores consistency across the available evidence.

## 3. Results

### 3.1. Mental Health, Wellbeing, and Quality of Life

#### 3.1.1. GRADE Evaluation

**Figure 1:**
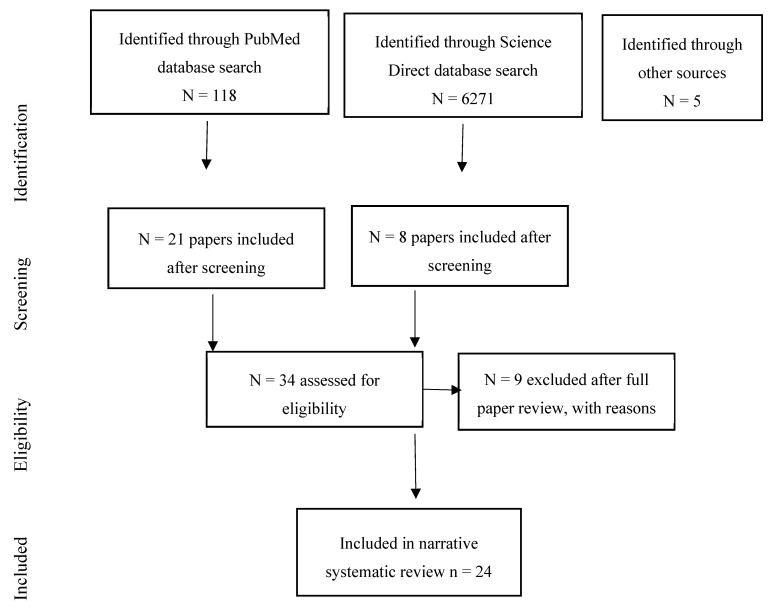
Flow chart showing the review process for the quality of life, wellbeing, and mental health papers.

The systematic review identified 29 studies of associations of environmental noise on mental health, wellbeing, and quality of life [19,20,21,22,23,24,25,26,27,28,29,30,31,32,33,34,35,36,37,38,39,40,41,42,43,44,45,46,47]. Two additional studies were identified from the searches conducted for cognition [48], and birth outcomes [49], respectively. The national UK Survey of Noise Attitudes 2014 [50] was also added, along with a paper from the NORAH study which had not been identified from the database searches [51]. Another study was identified from the recent Internoise 2019 conference [52] giving a total of 34 studies for consideration. Ten studies were excluded as they did not directly measure noise [20,21,24,25,26,27,35,37], or because no associations between noise exposure and mental health were reported in the paper [29,32] (see Appendix A.2). This left 24 studies for inclusion in the review. Figure 1 summarises the review process.

The studies were conducted in Belgium, Bulgaria, Canada, Finland, Germany, the Netherlands, New Zealand, Norway, South Korea, Sweden, and the United Kingdom. Most studies examined road traffic noise. The studies of adult mental health and wellbeing examined a range of outcomes including post-partum depression, medical diagnoses of depression and anxiety, medication use, symptom scales of mental health, wellbeing, and quality of life. For children the outcomes included the Strengths and Difficulties questionnaire [53], as well as symptom scores or diagnoses for inattention/ADHD (attention deficit hyperactivity disorder) and self or parental reports of wellbeing. There was evidence from longitudinal cohort studies for some outcomes (emotional and conduct disorders in children; medication intake; interview measures of depression and anxiety disorder) but most evidence was cross-sectional studies.

The detailed data extraction for these studies is shown in Table S1 (Supplementary Material). Many of the studies were rated as having unclear or high bias often because of low response rates or response rates not being stated. The GRADE evaluation for these papers is given in Table 7.

#### 3.1.2. Comparison of the Systematic Review Findings of the Review Undertaken for the WHO

The review carried out for the WHO on mental health, wellbeing, and quality of life [6] covered the evidence from a 10-year period, whereas the current review covers a four-year period. It is therefore prudent to consider whether the strength of the evidence identified within the WHO review is informative over and above the conclusions of the current review, which only covers a more limited timeframe. The key question is whether the studies identified in the current review would alter or strengthen the conclusions of the review undertaken for the WHO.

The conclusions from the review undertaken for the WHO for aircraft noise and mental health, wellbeing, and quality of life do not differ greatly in comparison with the conclusions of the current review. The current review was not able to reassess many of the outcomes for aircraft noise and mental health, wellbeing, and quality of life because of a lack of new studies. The current review suggests that the conclusions for the effect of aircraft noise on interview measures of depressive and anxiety disorders could be updated, as the evidence now suggests that there is low quality evidence for a harmful effect. This difference is attributable to the publication of several longitudinal studies since the review undertaken for the WHO, and the conclusion of the current review should be considered to stand.

The conclusions from the review undertaken for the WHO for road traffic noise and mental health, wellbeing, and quality of life do differ slightly in comparison with the conclusions of the current review. The review undertaken for the WHO concluded that there was very low-quality evidence for no effect of road traffic noise on interview measures of depressive and anxiety disorders, whereas the current review suggests there is now low quality evidence for a harmful effect. This difference can be attributed to an increase in longitudinal evidence since the review undertaken for the WHO, and the conclusion of the current review should be considered to stand. During the preparation of this paper, a further systematic review of road traffic noise effects on depression and anxiety [54] was published, which meta-analysed a wide range of measures of depression and anxiety measures (medication use, symptom reports, diagnoses), concluding that there was low quality evidence for a harmful effect. This conclusion agrees with the current systematic review.

For children and adolescents, a systematic review published whilst the current review was conducted [55] undertook a meta-analysis of three studies finding a harmful effect of road traffic noise on hyperactivity/inattention and total difficulties score of the Strengths and Difficulties Questionnaire [56] but no effect for conduct problems or emotional symptoms. The current review has concluded that there is low quality evidence for a harmful effect for emotional and conduct disorders, and hyperactivity, and very low-quality evidence for no effect for ADHD. However, the reviews differ in methodology, as Schubert et al., 2019 did not undertake the GRADE assessment but has the advantage of conducting meta-analyses albeit on a very small number of studies. All the reviews of the field of environmental noise effects on mental health identify the need for more studies that use similar outcomes and techniques [6,54,55,57]. At present, taken as a whole, the evidence suggests there are harmful effects of noise on mental health for children and adolescents: further studies will help to clarify whether this relationship holds for the wide variety of childhood mental health outcomes that have been investigated.

The review undertaken for the WHO concluded that there was very low quality evidence for no effect of road traffic noise on medication intake for the treatment of anxiety and depression assessing three studies, whereas the current review suggests there is very low quality evidence for an effect assessing two studies. However, the evidence supporting an effect comes only from one study and the three studies in the review undertaken for the WHO did not find an effect (With the exception of one study that found an effect in a sub-sample only.). Taken as a whole, the conclusion of the review undertaken for the WHO should be considered to stand until further evidence is forthcoming. Both reviews concluded that there is very low-quality evidence for no effect of road traffic noise on self-reported depression, anxiety, and psychological symptoms. The review undertaken for the WHO concluded that there was moderate quality evidence for a harmful effect of road traffic noise on emotional and conduct disorders in children and hyperactivity. The current review considers the evidence for these outcomes to be of low quality for a harmful effect, albeit based on far fewer studies. The conclusions of the review undertaken for the WHO should be considered to stand as the conclusion is drawn from a greater number of studies.

The conclusions from the review undertaken for the WHO for railway noise and mental health, wellbeing, and quality of life are little changed by the findings of the current review. The current review additionally suggests that there is low quality evidence for a harmful effect of railway noise on interview measures of depressive and anxiety disorders, which the review undertaken for the WHO did not assess due to a lack of evidence. The findings of the WHO assessment for railway noise should be considered to stand, with the addition of the finding for interview measures of depressive and anxiety disorders and for medication intake for the treatment of anxiety and depression.

The current review is additionally able to conclude that there is very low-quality evidence for no effect of wind turbine noise on self-reported quality of life or health which was not assessed in the review undertaken for the WHO.

### 3.2. Cancer

#### GRADE Evaluation

**Figure 2:**
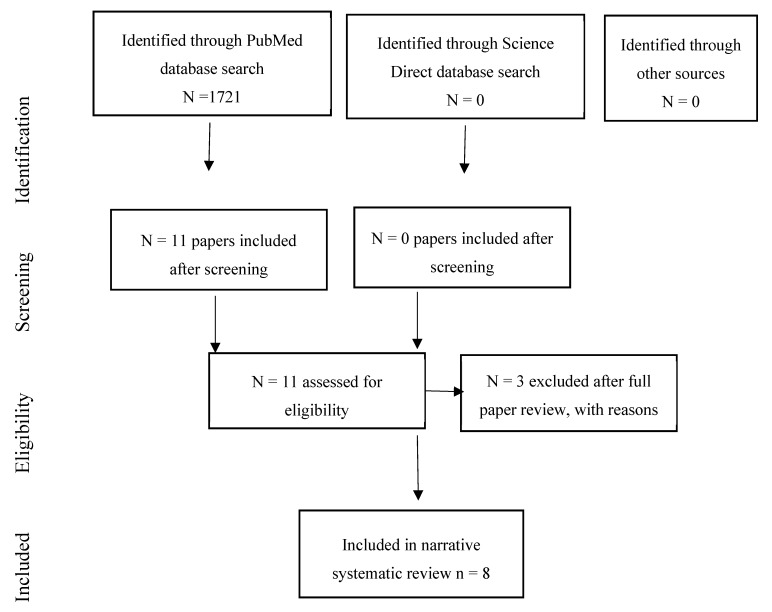
Flow chart showing the review process for the cancer papers.

The systematic review identified 11 studies of associations of environmental noise on cancer [12,13,58,59,60,61,62,63,64,65,66]. Three studies were excluded after data extraction [61,65,66] as they did not assess a cancer outcome per se or did not measure noise (see Appendix A.2). This left eight studies in the review. Figure 2 summarises the review process.

Seven of these studies were conducted in Denmark, with six out of eight studies being from large Danish Diet, Health, and Cancer longitudinal cohort study. The other studies were of a different Danish sample and a sample from Frankfurt, Germany. The studies consider the effects of noise on the incidence of a number of types of cancer, including breast cancer, colorectal cancer, prostate cancer, and non-Hodgkin’s lymphoma, as well as sub-types for some of the cancers. Some evidence is available assessing cancer at the population level, using established markers such as all-cause mortality from cancer (that is, cancer mortality for all cancers combined). The studies were longitudinal prospective cohort studies or case control studies. Most studies examined road traffic noise, but some studies also considered railway noise or aircraft noise. The detailed data extraction for these studies is shown in Table S2 (Supplementary Material). The studies were all individually rated as having low bias (Table 3). The GRADE evaluation for these papers is given in Table 8.

Cancer is an emerging health outcome in the field of noise and health. In future, it may be worth exploring the application of meta-analysis to the evidence for cancer, to estimate the association of noise with cancer across the studies. However, a few more studies per noise source and cancer outcome may be needed before this would be possible.

### 3.3. Dementia and other Neurodegenerative Outcomes

#### GRADE Evaluation

**Figure 3:**
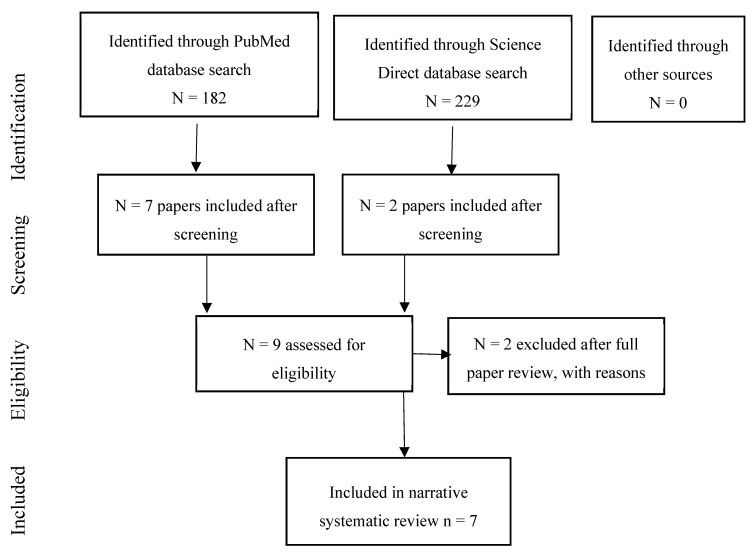
Flow chart showing the review process for the dementia papers.

The systematic review identified nine studies of associations of environmental noise on dementia and other neurodegenerative outcomes [14,15,67,68,69,70,71,72,73]. Two studies were excluded after data extraction as one did not did not measure noise and the other reported air pollution but not noise exposure [70,71] (see Appendix A.2). This left seven studies in the review. Figure 3 summarises the review process.

The studies were from European countries including Germany, Spain, Sweden, and the United Kingdom, with a mix of evidence from longitudinal cohort studies and longitudinal time-series studies. The studies examined road traffic noise and considered a range of outcomes including medical diagnoses of Parkinson’s disease, dementia, or Alzheimer’s disease, hospitalisations for dementia-related illnesses, as well as cognitive tests of dementia or dementia symptoms or precursors to dementia. Other neurodegenerative outcomes such as multiple sclerosis have been studied in a very limited number of studies. The detailed data extraction for these studies is shown in Table S3 (Supplementary Material). Only one study was rated as having low bias [14], with the other studies rated as having unclear or high bias (see Table 4). The GRADE evaluation for these papers is given in Table 9.

### 3.4. Birth and Reproductive Outcomes

#### 3.4.1. GRADE Evaluation

**Figure 4:**
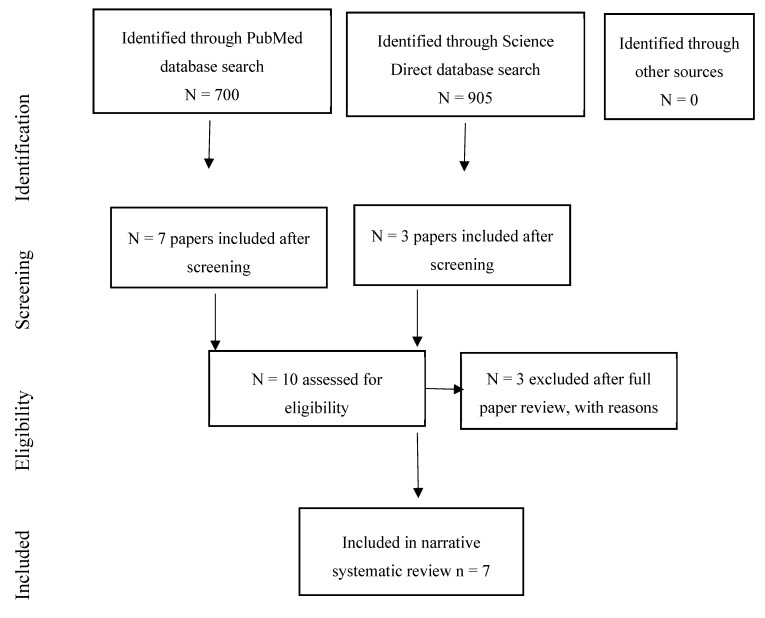
Flow chart showing the review process for birth outcome papers.

The systematic review identified ten studies of associations of environmental noise on birth and reproductive outcomes [49,74,75,76,77,78,79,80,81,82]. Three studies were excluded after data extraction (see Appendix A.2). One study was excluded as it reported noise exposure during pregnancy but no relevant health outcomes [79]. One study was about post-partum depression rather than a birth outcome for the infant, per se, so this paper was moved to the review for mental health, wellbeing and quality of life [49]. One study was excluded as it measured distance to road and not noise exposure, per se [80]. This left seven studies in the review. Figure 4 summarises the review process.

The studies were of samples from Austria/Italy, Canada, Denmark, Korea, Sweden, and the United Kingdom, with evidence from longitudinal and retrospective cohort studies. Most studies examined road traffic noise, with one study examining wind-turbine noise [82]. The studies considered a range of birth outcomes including pre-term birth, low birth weight, small for gestational age, as well as body mass index (BMI) in later childhood. One study examined the association between road noise and medically assessed male infertility [78] and another examined the association between road traffic noise and febrile seizures (full body convulsions caused by high fever in childhood) in childhood [77]. One study examined congenital abnormalities at birth [76].

The detailed data extraction for these studies is shown in Table S4 (Supplementary Material). The studies were individually all rated as having low bias (Table 5). The GRADE evaluation for these papers is given in Table 10.

#### 3.4.2. Comparison of the Systematic Review Findings of the Review Undertaken for the WHO

The current review identified no papers of aircraft noise, therefore, we consider the conclusions of the review undertaken for the WHO [4] regarding aircraft noise and birth outcomes to stand.

For road noise and birthweight, the findings of the current review differ from those of the review undertaken for the WHO. The review undertaken for the WHO concluded based on the findings of eight studies that there was ‘low quality evidence for an association of road traffic noise on low birth weight’, whereas the current review concludes that there is high quality evidence for no effect of road traffic noise on birthweight, based on the findings of two longitudinal studies. In examining the findings of the review undertaken for the WHO, despite the conclusion drawn the evidence was quite mixed with only some studies showing an association. The GRADE used in both reviews is precautionary, in that, if some but not all evidence shows an effect then the conclusion will indicate that there is an effect. During the preparation of this paper a further systematic review of road traffic noise effects on birth outcomes [83] was published. Using meta-analysis that paper found a moderate effect of road traffic noise on low birthweight (when measured continuously) but low quality evidence for no effect for studies that examined low birth weight categorically [83]. Overall, the two most recent reviews agree to some extent, but not entirely, that there is a no harmful effect of road traffic noise on birthweight. Overall, the findings of the current review add to the equivocality of the evidence regarding birth weight. For the UK context, the evidence from the large-scale study by Smith, Fecht, Gulliver, Beevers, Dajnak, Blangiardo, Ghosh, Hansell, Kelly, Anderson and Toledano [74] is compelling and should perhaps inform the conclusion that at present for the UK context it is appropriate to consider that there is no effect of road traffic noise on birth weight. The recent systematic review [83] concluded there was low quality evidence for no effect of road traffic noise small for gestational age, mirroring the conclusion of the current review.

### 3.5. Cognition

#### 3.5.1. GRADE Evaluation

**Figure 5:**
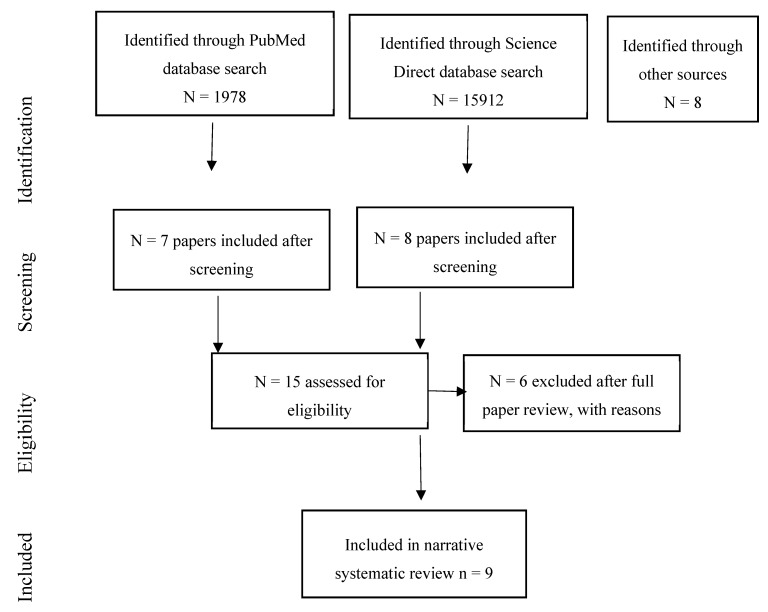
Flow chart showing the review process for the cognition paper.

The systematic review identified nine studies of associations of environmental noise on cognition [39,48,84,85,86,87,88,89,90]. Studies examined child and adult samples. A further three studies from the NORAH study, known to the authors were added: one that had not been identified by the systematic searches [51], as well as three conference papers [91,92,93]. Two other studies known to the authors were also added [94,95], along with another recent conference paper [93]. Six studies were excluded after data extraction (Appendix A.2) which included two studies which reported experimental studies [84,85]; one that reported on mental health and not cognition and had already been identified in the search for mental health [39]; one which did not report on noise exposure per se [89], and another study which reported an ADHD outcome, which was moved to the mental health review [48]. One study reported on attitudes to noise within the school and did not report a cognitive outcome [90]. This left nine studies in the final review. Figure 5 summarises the review process.

The studies were from Germany, Greece, Spain, South Africa and the United States, with a mix of evidence from longitudinal cohort studies and cross-sectional studies. The studies considered a range of cognitive outcomes including cognitive testing of reading and mathematics for children, as well as cognitive testing of adults. One study reported on an observational study of student distraction by aircraft noise during class: this is a potentially weaker measure of cognition but is included in the review given the limited studies available. One study examined the effect of road traffic noise at school on developmental trajectories for working memory and attention. The available studies were of road traffic noise and aircraft noise exposure. The detailed data extraction for these studies is shown in Table S5 (Supplementary Material). The GRADE evaluation for these papers is given in Table 11.

#### 3.5.2. Comparison of the Systematic Review Findings of the Review Undertaken for the WHO

The conclusions of the review undertaken for the WHO [5] differ to those of the current review. For reading comprehension, the review undertaken for the WHO concluded that there was “moderate quality evidence for an effect of aircraft noise on children’s reading and oral comprehension” and “low quality evidence for no substantial effect of road traffic noise on children’s reading and oral comprehension”. The current review finds very low-quality evidence for an effect of aircraft noise and road traffic noise on children’s reading comprehension. However, this reflects the smaller number of studies in the current review, despite the inclusion of methodologically robust studies such as NORAH [51]. This is because methodologically weaker studies included within the body of evidence impact on the GRADE process and result in downgrading. For reading comprehension, the review undertaken for the WHO included 14 studies of aircraft noise and 2 studies of road traffic noise, whereas the current review included four studies of aircraft noise and one study of road traffic noise. The additional aircraft noise studies identified in the past four years and the conclusions drawn from their review would not conflict with conclusions of the review undertaken for the WHO. The conclusions of the review undertaken for the WHO should be considered to stand in light of the current review’s conclusions.

For road traffic noise the conclusion of the review undertaken for the WHO was based on two studies showing no effect on reading comprehension (both of which reported on the RANCH study) and the current review now identifies one additional paper that shows an effect but is not methodologically robust. Taking the precautionary approach, we could recommend the finding of the current review that there is ‘very low-quality evidence for an effect of road traffic noise on reading comprehension’. However, this conclusion has to be tempered by the high risk of bias for the one study on which the conclusion was based which did not clearly report how children were recruited and did not adjust the finding for confounding factors, versus the findings of the large-scale methodologically robust RANCH study which has clear relevance of the UK context (as one of the samples was from around London Heathrow airport). At this stage, until further evidence is available, it would be prudent to rely on the conclusions of the review undertaken for the WHO.

The review undertaken for the WHO concluded that there was low quality evidence for no effect of road traffic noise on executive function/working memory based on five cross-sectional studies. The current review concludes that there is low quality evidence for an effect of road traffic noise on working memory in children based on one longitudinal study. Comparing the conclusions therefore involves weighing up a few cross-sectional studies versus one longitudinal study: as a precautionary approach the conclusion of the current review is put forward as an update to the conclusion of the review undertaken for the WHO.

The review undertaken for the WHO concluded that there was very low-quality evidence for no effect of road traffic noise on attention based on five cross-sectional studies. The current review concludes that there is low quality evidence for an effect of road traffic noise on attention in children based on one longitudinal study. Comparing the conclusions therefore involves weighing up a few cross-sectional studies versus one longitudinal study: as a precautionary approach the conclusion of the current review is put forward as an update to the conclusion of the review undertaken for the WHO.

## 4. Discussion

### 4.1. Recommendations for Consideration by the IGCB(N)

This systematic review has assessed the quality of the evidence across the available studies for aircraft noise exposure, road traffic noise exposure, and railway noise exposure on a range of health outcomes including mental health, wellbeing, and quality of life; cancer; dementia and other neurodegenerative disorders; birth and reproductive outcomes; and cognition. The scope of this review included a wide-range of environmental noise sources, yet the available evidence predominantly related to road traffic noise, aircraft noise, and railway noise. There were very few studies of the other environmental noise sources including wind turbine noise, building services noise, ventilation noise, neighbour noise, industrial noise, leisure noise or combined noise. The health effects of these noise sources remain unquantified.

The review was undertaken to consider the implications of the findings for the IGCB (N) and has used the GRADE assessment methodology to determine the strength of the evidence for the evidence-base published since the publication of the systematic reviews undertaken for the WHO. Given the shorter time-frame for the current systematic reviews than the systematic reviews undertaken for the WHO, an assessment has been made as to whether the findings of the current review would alter or strengthen the conclusions of the WHO reviews. Table 12, Table 13 and Table 14 provide a summary of the conclusions of the systematic reviews undertaken for the WHO with the conclusions of the current systematic review.

The systematic reviews were undertaken to consider the implications of the findings for the IGCB (N). The systematic reviews have considered whether here is a harmful effect or no effect of the environmental noise exposure on the various health outcomes and these recommendations are provided below. Where no effect was identified, no recommendation is provided, as there is no need to quantify the effect of the exposure on the health outcome. Given the breadth of outcomes available for most of the health and cognitive outcomes examined in the review, recommendations were made on the basis of the strongest epidemiological outcomes, where possible, so for example, the incidence of dementia or depression, rather than assessments of symptoms. As meta-analyses have not been undertaken, recommendations regarding the evidence follow the previous IGCB (N) approaches, in terms of recommending relationships for a particular noise source and outcome. Previous IGCB (N) recommendations have also applied relationships from one noise source, to estimate effects for a different noise source, where evidence for the noise source was not yet available. This approach was also taken here.

A large body of evidence was identified relating to environmental noise effects on mental health, but this is an area that is still beset by some poor quality studies for many outcomes (Table 12). In terms of mental health, wellbeing and quality of life evidence from UK studies is mixed and limited to self-reported health, quality of life and wellbeing measures. The national Survey of Noise Attitudes 2014 failed to find associations between aircraft noise (LAeq 16h) and self-reported health or the Warwick Edinburgh Mental Wellbeing Scale, although it did find associations for these outcomes with noise annoyance [50]. A UK study using census data for people living around 17 airports and a measure of wellbeing, found that day-time aircraft noise was associated with wellbeing [46]: no association was found between night-time aircraft noise exposure and wellbeing. Another study from the United Kingdom using census data from around Belfast Airport failed to find an association between aircraft noise and self-reported mental health assessed as “an emotional, psychological or mental health condition (such as depression or schizophrenia)” [47]. There is a need for longitudinal surveys in the UK that assess symptoms and interview measures of depression and anxiety, as well as self-reported depression, anxiety, and psychological symptoms.

However, considering the past reviews [6,57] alongside the current review, it can be concluded that there is enough evidence for ERFs between noise (road, railway, aircraft) and adult and childhood mental health. Following previous IGCB(N) approaches it would be possible, for example, to use the NORAH study for adult mental health [23] which assessed the incidence of depression and anxiety. However, as the aircraft ERF from the NORAH study is not reliable at higher exposures it may be appropriate to use the road ERF from this study for all noise sources until further ERFs become available. For children, several methodologically robust studies are available that could also be used such as those identified in the review undertaken for the WHO for road traffic noise and railway noise such as Dreger, Meyer, Fromme and Bolte [26] which examines incident mental health symptoms. This should not, however, be applied for aircraft noise, as neither the WHO review or this review found evidence for a harmful effect for aircraft noise.

Whilst wellbeing as a concept has risen in popularity in recent years, the review identified few studies of environmental noise and wellbeing. This review concluded that there was very low-quality evidence for an effect of aircraft noise on wellbeing (Table 12). As for mental health, adopting a precautionary approach it would be possible to use a study from this evidence base, for example Lawton and Fujiwara [46], to estimate noise effects on wellbeing.

No recommendation is made for quality of life, as both this review and the review undertaken for the WHO concluded that there was very low-quality evidence for no effect of aircraft noise on self-reported health or quality of life. The WHO review came to the same conclusion for road traffic noise but did find very low quality evidence for a harmful effect for railway noise (Table 12). This is a research area that should be monitored to see if methodologically robust evidence for a harmful effect becomes available in the next few years. It is of great frustration to local communities that the evidence base in this area is not more robust or consistent, as quality of life effects are often reported and are a keen concern for noise exposed communities.

This review is one of the first to consider the emerging body of evidence for environmental noise effects on cancer. Overall, given the number of studies available, the evidence is quite convincing for effects of aircraft noise, road traffic noise, and railway noise on some cancer outcomes. However, no evidence is yet available for the UK. For estimating effects at the population level, it would be useful to have evidence or an ERF for a relevant population-level cancer outcome, such as all-cause mortality from cancer. At present, the data only supports an effect for some types of cancer and different sub-types of the same cancer show different associations. At this point, given that most of the evidence currently comes from one Danish birth cohort it is worth keeping a watching brief on this area, as further evidence becomes available which considers wider population measures of cancer.

The review has concluded that there is low quality evidence for no effect of road traffic noise on the incidence of vascular dementia. Evidence is available from a large-scale methodologically robust UK study which found that the association between road noise and an incidence diagnosis of dementia became non-significant after adjustment for air pollution [14]. Therefore, no study is recommended to the IGCB (N) for this health outcome. There is very limited evidence relating to other neurological conditions and no studies of incidence, to date.

Overall, evidence for effects on birth and other reproductive outcomes remains equivocal, with most studies showing no association (for the UK context, the evidence from the large-scale study by Smith, Fecht, Gulliver, Beevers, Dajnak, Blangiardo, Ghosh, Hansell, Kelly, Anderson and Toledano [74]) is compelling and informs the conclusion that at present for the UK it is appropriate to conclude that there is no effect of road traffic noise on birth weight and to apply this finding to other noise sources (Table 13).

Evidence from the methodologically robust NORAH study [51] confirms the findings of the UK-relevant RANCH study in terms of effects on children’s reading comprehension [96]. The evidence is certainly strong enough to support applying the aircraft noise ERFs from RANCH or NORAH to estimate effects of environmental noise on children’s reading comprehension (Table 14). However, the RANCH study did not find an effect of road traffic noise on reading comprehension, which suggests that the aircraft noise relationship should not be applied for road traffic noise. Studies of adulthood cognition are starting to emerge, particularly in relation to the development of dementia in later-life. Given the overlap in the evidence to date, this should perhaps be considered in relation to vascular dementia as an outcome and not cognition.

### 4.2. Strengths and Limitations

Strengths of this systematic review include the addition of emerging health outcomes not previously reviewed for the evidence reviews undertaken for the WHO including cancer, and dementia and other neurodegenerative outcomes, as well as narrative updates to the reviews for cognition and mental health, wellbeing, and quality of life. The review was carried out by one of the authors of the reviews undertaken for the WHO on cognition and mental health, wellbeing, and quality of life ensuring consistency in methodological approach across the reviews. The current review has been able to consider the impact of large-scale longitudinal papers published since the reviews undertaken for the WHO including evidence from the NORAH study and other large scale studies [23,51,74].

Limitations of the reviews include the relatively short-time frame covered by the reviews; restrictions to papers published in English and the use of only two databases for the searches (PubMed and Science Direct) due to time-constraints for this rapid review. The scope of this review was to examine the evidence for an effect for each measure of the health outcome individually, rather than combining across wider categorisations, as undertaken by the recent systematic review and meta-analysis of road traffic noise effects on depression and anxiety [54]. However, despite differing methodological approaches, the two recent reviews come to the same conclusion. As identified in the reviews undertaken for the WHO, when looking at specific noise sources and specific outcomes there are often relatively few papers available and relatively few longitudinal papers. The evidence relating to environmental noise effects on vascular dementia includes studies that examine the short-term association between road traffic noise exposure and emergency hospitalisations for dementia [68,69]. The authors of these papers speculate that short term exposure to noise may lead to an exacerbation of symptoms of a mental disease such as dementia, which might lead to emergency admission to hospital of persons already suffering from the disease. However, we consider that this may be a biased measure of dementia. Evidence suggests that for dementia patients who undergo emergency hospitalisation in the UK, the primary cause is often not their dementia diagnosis per se but attributed to other causes such as syncope (fainting), collapse, bronchopneumonia, urinary tract infection, and dehydration [97]. There are many other factors that are likely to influence emergency hospitalisation for dementia patients, making the hypothesis relating to short-term noise exposure seem unlikely. A further two studies from this Spanish research team also assess the short-term associations between road traffic noise exposure and emergency hospitalisation for Parkinson’s disease and multiple sclerosis [72,73], as well as health care use for Parkinson’s disease [72].

A further limitation lies in applying the GRADE methodology to studies of environmental epidemiology, per se. When used in studies of environmental noise and health, the GRADE methodology often results in downgrading of the evidence and very rarely in upgrading of the evidence. There are several reasons for this. Firstly, there are often inconsistent findings across the body of evidence, e.g., the evidence is often a mix of studies that do and do not show an association, which will result in downgrading. This is often the case where there are few studies available for a noise source and a health outcome but is also found where there are a larger number of studies, as the likelihood of inconsistency increases the greater the number of studies that are available. If only one study is available for a specific noise source and a health outcome then consistency cannot be assessed and the evidence is downgraded automatically (this matches the approach used in the systematic reviews undertaken for the WHO). Across the review, there are very few instances where the quality of the evidence does not get downgraded for inconsistency, perhaps reflecting a weakness of the GRADE process when applied to epidemiological rather than clinical research studies. Secondly, whilst the assessment of the overall quality of evidence reflects the strengths and weaknesses introduced by inclusion of all the studies identified in the search, the weaknesses can end up carrying a greater weight in the assessment. If methodologically weaker studies are included within the body of evidence, it does not really matter how methodologically robust the ‘best’ study is, as the other studies will result in a downgrading of the evidence. Thirdly, in terms of upgrading the evidence, whilst recent epidemiological studies typically adjust for a wide-range of relevant confounders and covariates, it can be very difficult to conclude with confidence that adjustment for further factors may not alter the effect. It is also worth noting, some study designs adjust for a limited number of covariates and confounders. For example, ecological studies such as a study of hospital admissions for a specific health outcome within an entire population usually cannot adjust for relevant socioeconomic or other health-related covariates at the individual level: instead, they only adjust for area-level socioeconomic and other health-related covariates, which means that confounding cannot be ruled out.

## 5. Conclusions

The evidence for effects of environmental noise such as road traffic noise and railway noise has increased for some health outcomes since the publication of the WHO evidence reviews. In particular, there is now low-quality evidence for a harmful effect of road traffic noise on medication use and interview measures of depression and anxiety. However, many other conclusions from the WHO evidence reviews remain unchanged. There is low-quality evidence for a harmful effect of road traffic noise on some cancer outcomes. The conclusions of this review are limited by the low number of studies for many health and cognitive outcomes. The low-quality evidence across studies for noise effects for some outcomes does not necessarily mean that there are no effects: rather, that more robust studies and a greater number of studies are required. The quantification of health effects for other noise sources such as wind turbine noise, building services noise, ventilation noise, neighbour noise, industrial noise, leisure noise, or combined noise remains a research priority.

## Figures and Tables

**Table 1:** Summary of health outcomes and temporal scope for the systematic review.

WP2: Health Outcome	Temporal Scope of Review
Cognition	June 2015 to March 2019
Dementia and other neurodegenerative diseases	January 2014 to March 2019
Mental health, quality of life, and wellbeing	October 2015 to March 2019
Birth and reproductive outcomes	January 2017 to March 2019
Cancer	January 2014 to March 2019

**Table 2:** Mental health, wellbeing and quality of life: risk of bias.

Reference	Bias due Exposure Assessment	Bias due to Confounding	Bias due to Selection of Participants	Bias due to Health Outcome Assessment	Bias due to Not Blinded Outcome Assessment	Total Risk of Bias
Weyde, Envt Health, 2017	Low	Low	High	Unclear	Low	High
Feder et al., Environ Res, 2015	Low	Low	Low	Low	Low	Low
Seidler et al., Environ Res, 2017	Unclear	Low	Low	Low	Low	Unclear
Welch et al., Noise Health, 2018	Low	Unclear	Low	Low	Low	Unclear
Klatte et al., Environ & Behavior, 2016	Low	Low	Low	Low	Low	Low
Dzhambov et al., Environ Res, 2018a	Low	Low	Unclear	Low	Low	Unclear
Generaal et al., Psychol Med, 2019	Low	Low	High	Low	Low	Unclear
Dzhambov et al., Environ Res, 2018b	Low	Low	Unclear	Low	Low	Unclear
Dzhambov et al., Environ Int., 2017	Low	Low	Unclear	Low	Low	Unclear
Zock et al., Environ Int., 2018	Low	Low	Low	Low	Low	Low
Lim et al., Noise Health, 2018	Low	Low	High	Low	Low	Unclear
Forns et al., Enviro Health Perspectives, 2016	Low	Low	Low	Low	Low	Low
He et al., Environ Res., 2019	Low	Low	Unclear	Low	Low	Unclear
Civil Aviation Authority, 2017	Low	High	Low	Low	Low	Unclear
Van Aart et al., Environ Int., 2018	Low	Low	High	Low	Low	Unclear
Klompmaker et al., Environ Int., 2019	Low	Low	Unclear	Low	Low	Unclear
Okokon et al., Environ Int., 2018	Low	Low	Unclear	Low	Low	Unclear
Oiamo et al., Soc Sci Med., 2015	Low	High	Unclear	Low	Low	Unclear
Leijssen et al., IJERPH, 2019	Low	Low	Low	Low	Low	Low
Zijlema et al., Int. J Hygiene E Health., 2015	Low	Low	Unclear	Low	Low	Unclear
Wallas et al., Int. J Hygiene E Health., 2018	Low	Unclear	High	Low	Low	Unclear
Lawton et al., Transport Res Part D., 2016	Low	Low	Low	Low	Low	Low
Wright et al., Environ Health., 2018	Low	Low	Low	Low	Low	Low
Zijlema et al., Internoise., 2019	Low	Low	Unclear	Low	Low	Unclear

**Table 3:** Cancer: risk of bias.

Reference	Bias due Exposure Assessment	Bias due to Confounding	Bias due to Selection of Participants	Bias due to Health Outcome Assessment	Bias due to Not Blinded Outcome Assessment	Total Risk of Bias
Andersen et al., Lynge Breast Cancer Res., 2018	Low	Low	Low	Low	Low	Low
Hegewald et al., Scandinavian J Work Envt Health, 2017	Low	Low	Low	Low	Low	Low
Roswall et al., Environ Research, 2016	Low	Low	Low	Low	Low	Low
Roswall, et al., Cancer, Causes & Control, 2017	Low	Low	Low	Low	Low	Low
Roswall et al., PloS One, 2015	Low	Low	Low	Low	Low	Low
Roswall et al., PloS One, 2017	Low	Low	Low	Low	Low	Low
Sorensen et al., I J of Cancer, 2014	Low	Low	Low	Low	Low	Low
Sorensen et al., Environmental Research, 2015	Low	Low	Low	Low	Low	Low

**Table 4:** Dementia and other neurodegenerative outcomes: risk of bias.

Reference	Bias due Exposure Assessment	Bias due to Confounding	Bias due to Selection of Participants	Bias due to Health Outcome Assessment	Bias due to Not Blinded Outcome Assessment	Total Risk of Bias
Andersson et al., Environmental Research, 2018	Low	Low	Unclear	Low	Low	Unclear
Carey et al., BMJ Open, 2018	Low	Low	Low	Low	Low	Low
Culqui et al., Science of Total Environment, 2017	Low	High	Low	Low	Low	Unclear
Linares et al., Environ Res., 2017	Low	High	Low	Low	Low	Unclear
Tzivian et al., Environmental Health Perspectives, 2016	Low	Low	Unclear	Low	Low	Unclear
Diaz et al., Gac Sanit, 2018	Low	High	Low	Low	Low	Unclear
Carmona et al., Science of Total Environment, 2017	Low	High	Low	Low	Low	Unclear

**Table 5:** Birth and reproductive outcomes: risk of bias.

Reference	Bias due Exposure Assessment	Bias due to Confounding	Bias due to Selection of Participants	Bias due to Health Outcome Assessment	Bias due to Not Blinded Outcome Assessment	Total Risk of Bias
Hjortebjerg et al., Scand J Work Environ Health, 2018	Low	Low	Low	Low	Low	Low
Min & Min, Environ Pollut., 2017	Low	Low	Low	Low	Low	Low
Pedersen et al., Environ Res., 2017	Low	Low	Low	Low	Low	Low
Smith et al., BMJ, 2017	Low	Low	Low	Low	Low	Low
Wallas et al., Environ Res., 2019	Low	Low	Low	Low	Low	Low
Poulsen et al., Environ Res., 2018	Low	Low	Low	Low	Low	Low
Dzhamov et al., Sci Tot Envt., 2019	Low	Low	Low	Low	Low	Low

**Table 6:** Cognition: risk of bias.

Reference	Bias due Exposure Assessment	Bias due to Confounding	Bias due to Selection of Participants	Bias due to Health Outcome Assessment	Bias due to Not Blinded Outcome Assessment	Total Risk of Bias
Papanikolaou et al., Int J Adolesc Med Health, 2015	Unclear	High	Unclear	Low	Low	High
Seabi et al., J Expo Sci Environ Epidemiol., 2015	Low	Low	High	Low	Low	Unclear
Tzivian et al., Environ Health Perspectives, 2016	Low	Low	Low	Low	Low	Low
Tzivian et al., J Toxicol Environ Health A, 2017	Low	Low	Low	Low	Low	Low
Klatte et al., Environ & Behavior, 2016	Low	Low	Low	Low	Low	Low
Spilski et al., ICBEN, 2017	Low	Low	Low	Low	Low	Low
Spilski et al., Internoise, 2017	Low	Low	Low	Low	Low	Low
Eagen et al., Transport Research Board, 2017	Low	High	High	High	Unclear	High
Foraster et al., Internoise, 2017	Low	Low	Unclear	Low	Low	Unclear

**Table 7:** Summary of the strength of the evidence for mental health, wellbeing, and quality of life.

Mental Health, Wellbeing and Quality of Life	Environmental Noise Exposure			
Domain	Aircraft Noise: Quality of Evidence and Assessment of Effect	Road Traffic Noise: Quality of Evidence and Assessment of Effect	Railway Noise: Quality of Evidence and Assessment of Effect	Wind Turbine Noise: Quality of Evidence and Assessment of Effect
Self-reported quality of life or health	Very low quality—no effect (4)	n.a.	n.a.	Very low quality—no effect (1)
Self-reported depression, anxiety, and psychological symptoms	n.a.	Very low quality—no effect (7)	Very low quality—no effect (1)	n.a.
Interview measures of depressive and anxiety disorders	Low quality—harmful effect (2)	Low quality—harmful effect (4)	Low quality—harmful effect (3)	n.a.
Wellbeing	Very low quality—harmful effect (3)	n.a.	n.a.	n.a.
Emotional and conduct symptoms in children	n.a.	Low quality—harmful effect (3)	n.a.	n.a.
Hyperactivity	n.a.	Low quality—harmful effect (3)	n.a.	n.a.
Cortisol in children	n.a.	Very low quality—harmful effect (1)	n.a.	n.a.
Medication intake for the treatment of anxiety and depression	n.a.	Very low quality—harmful effect (2)	Very low quality—harmful effect (1)	n.a.
ADHD in children	n.a.	Very low quality—no effect (1)	n.a.	n.a.

n.a. no studies available to evaluate (n) number of papers evaluated.

**Table 8:** Summary of the strength of the evidence for cancer.

Cancer	Environmental Noise Exposure			
Domain	Aircraft Noise: Quality of Evidence and Assessment of Effect	Road Traffic Noise: Quality of Evidence and Assessment of Effect	Railway Noise: Quality of Evidence and Assessment of Effect	Wind Turbine Noise: Quality of Evidence and Assessment of Effect
Cancer mortality	n.a.	High quality—no effect (2)	n.a.	n.a.
Incidence of breast cancer	Low quality—harmful effect (1)	Low quality—harmful effect (3)	Low quality—harmful effect (2)	n.a.
Incidence of colorectal cancer	n.a.	Low quality—harmful effect (1)	Low quality—no effect (1)	n.a.
Incidence of prostate cancer	n.a.	Low quality—no effect (1)	Low quality—no effect (1)	n.a.
Incidence of non-Hodgkin lymphoma	n.a.	Low quality—harmful effect (1)	n.a.	n.a.

n.a. no studies available to evaluate (n) number of papers evaluated.

**Table 9:** Summary of the strength of the evidence for dementia and other neurodegenerative outcomes

Dementia and Other Neurodegenerative Outcomes	Environmental Noise Exposure
Domain	Road Traffic Noise: Quality of Evidence and Assessment of Effect
Incidence of vascular dementia	Low quality—no effect (2)
Dementia related emergency admissions	Very low quality—harmful effect (2)
Cognitive assessment of dementia symptoms	Very low quality—harmful effect (1)
Multiple sclerosis related emergency admissions	Very low quality—harmful effect (1)
Parkinson’s Disease emergency admissions	Very low quality—harmful effect (1)
Parkinson’s Disease healthcare	Very low quality—harmful effect (1)
n.a. no studies available to evaluate (n) number of papers evaluated	

**Table 10:** Summary of the strength of the evidence for birth and reproductive outcomes.

Birth and Reproductive Outcomes	Environmental Noise Exposure			
Domain	Aircraft Noise Quality of Evidence and Assessment of Effect	Road Traffic Noise Quality of Evidence and Assessment of Effect	Railway Noise Quality of Evidence and Assessment of Effect	Wind Turbine Noise Quality of Evidence and Assessment of Effect
Low birth weight	n.a.	High quality—no effect (3)	Very low quality—no effect (1)	Moderate quality—no effect (1)
Pre-term birth	n.a.	Moderate quality—no effect (1)	n.a.	Moderate quality—no effect (1)
Small for gestational age	n.a.	Moderate quality—no effect (2)	Very low quality—no effect (1)	Moderate quality—no effect (1)
Congenital abnormalities	n.a.	Low quality—no effect (1)	n.a.	n.a.
Febrile seizures	n.a.	Low quality—harmful effect (1)	n.a.	n.a.
Male fertility	n.a.	Low quality—harmful effect (1)	n.a.	n.a.

n.a. no studies available to evaluate (n) number of papers evaluated.

**Table 11:** Summary of the strength of the evidence for cognition

Cognition	Environmental Noise Exposure			
Domain	Aircraft Noise: Quality of Evidence and Assessment of Effect	Road Traffic Noise: Quality of Evidence and Assessment of Effect	Railway Noise: Quality of Evidence and Assessment of Effect	Wind Turbine Noise: Quality of Evidence and Assessment of Effect
Reading comprehension	Very low quality—harmful effect (4)	Very low quality—harmful effect (1)	n.a.	n.a.
Mathematics	n.a.	Very low quality—harmful effect (1)	n.a.	n.a.
Working memory	n.a.	Low quality—harmful effect (1)	n.a.	n.a.
Attention	n.a.	Low quality—harmful effect (1)	n.a.	n.a.
Student distraction	Very low quality—harmful effect (1)	n.a.	n.a.	n.a.
Assessment of adult cognition	n.a.	Very low quality—harmful effect (2)	n.a.	n.a.

n.a. no studies available to evaluate (n) number of papers evaluated.

**Table 12:** Comparison of the strength of the evidence for the WHO 2018 and the current review for aircraft, road and railway noise and mental health, wellbeing and quality of life.

Outcome	WHO Clark & Paunovic 2018	Current Review
	**Aircraft noise**	
Self-reported quality of life or health	Very low quality—no effect	Very low quality—no effect (A similar assessment of very low quality evidence for no effect of wind turbine noise on self-reported quality of life or health was also found in the current review. This was not found in the WHO review.)
Medication intake for treatment of anxiety and depression	Very low quality—harmful effect	n.a.
Self-reported depression, anxiety and psychological symptoms	n.a.	n.a.
Interview measures of depressive and anxiety disorders	Very low quality—harmful effect	Low quality—harmful effect
Emotional and conduct disorders in children	Low quality—no effect	n.a.
Hyperactivity	Low quality—harmful effect	n.a.
Wellbeing	Not evaluated in the review	Very low quality—harmful effect
	**Road noise**	
Self-reported quality of life or health	Low quality—no effect	n.a.
Medication intake for treatment of anxiety and depression	Very low quality—no effect	Very low quality—harmful effect
Self-reported depression, anxiety and psychological symptoms	Very low quality—no effect	Very low quality—no effect
Interview measures of depressive and anxiety disorders	Very low quality—no effect	Low quality—harmful effect
Emotional and conduct disorders in children	Moderate quality—harmful effect	Low quality—harmful effect
Hyperactivity in children	Moderate quality—harmful effect	Low quality—harmful effect
Cortisol in children	n.a.	Very low quality—harmful effect
Wellbeing	Not included in this review	n.a.
ADHD in children	Not included in this review	Very low quality—no effect
	**Railway noise**	
Self-reported quality of life or health	Low—harmful effect	n.a.
Medication intake for treatment of anxiety and depression	n.a.	Very low quality—harmful effect
Self-reported depression, anxiety and psychological symptoms	n.a.	Very low quality—no effect
Interview measures of depressive and anxiety disorders	n.a.	Low quality—harmful effect
Emotional and conduct disorders in children	Moderate quality—harmful effect	n.a.
Hyperactivity	Moderate quality—no effect	n.a.
Wellbeing	n.a.	n.a.

n.a.—no studies available to evaluate.

**Table 13:** Comparison of the strength of the evidence for the WHO 2018 and the current review for aircraft, road and railway noise, and birth and reproduction.

Outcome	WHO Clark and Paunovic 2018	Current Review
	**Aircraft noise**	
Low birth weight	Very low quality—no effect	n.a.
Pre-term birth	Very low quality—no effect	n.a.
Small for gestational age	n.a.	n.a.
Cognitive abnormalities	Very low quality—no effect	n.a.
Febrile seizures	n.a.	n.a.
Male infertility	n.a.	n.a.
	**Road noise**	
Low birth weight	Low quality—no effect	High quality—no effect
Pre-term birth	Low birth weight	Moderate quality—no effect
Small for gestational age	Low birth weight	Moderate quality—no effect
Cognitive abnormalities	n.a.	Low quality—no effect
Febrile seizures	n.a.	Low quality—harmful effect
Male infertility	n.a.	Low quality—harmful effect
	**Railway noise**	
Low birth weight	n.a.	Very low quality—no effect
Pre-term birth	n.a.	n.a.
Small for gestational age	n.a.	Very low quality—no effect
Cognitive abnormalities	n.a.	n.a.
Febrile seizures	n.a.	n.a.
Male infertility	n.a.	n.a.

**Table 14:** Comparison of the strength of the evidence for the WHO 2018 and the current review for aircraft, road, and railway noise and cognition.

Outcome	WHO Clark and Paunovic 2018	Current Review
	**Aircraft noise**	
Reading comprehension	Moderate quality—harmful effect	Very low quality—harmful effect
Mathematics	n.a.	n.a.
Working memory	Very low quality—no effect	n.a.
Attention	Low quality—no effect	n.a.
Student distraction	n.a.	Very low quality—harmful effect
Assessment of adult cognition	n.a.	n.a.
Standardized assessment tests	Moderate quality—harmful effect	n.a.
Long-term and short-term memory	Moderate quality—harmful effect	n.a.
	**Road noise**	
Reading comprehension	Very low quality—no effect	Very low quality—harmful effect
Mathematics	n.a.	Very low quality—harmful effect
Working memory	Low quality—no effect	Low quality—harmful effect
Attention	Very low quality- no effect	Low quality—harmful effect
Student distraction	n.a.	n.a.
Assessment of adult cognition	n.a.	Very low quality—harmful effect
Standardized assessment tests	Very low quality- harmful effect	n.a.
Long-term and short-term memory	Very low quality- harmful effect	n.a.
	**Railway noise**	
Reading comprehension	n.a.	n.a.
Mathematics	n.a.	n.a.
Working memory	n.a.	n.a.
Attention	Very low quality—no effect	n.a.
Student distraction	n.a.	n.a.
Assessment of adult cognition	n.a.	n.a.
Standardized assessment tests	Moderate quality—harmful effect	n.a.
Long-term and short-term memory	Very low quality—harmful effect	n.a.

## Supplementary Materials

The following are available online at https://www.mdpi.com/1660-4601/17/2/393/s1, Table S1: Mental health, wellbeing and quality of life extraction table; Table S2: Cancer extraction table; Table S3: Dementia and other neurodegenerative outcomes extraction table; Table S4: Birth and reproductive outcomes extraction table; Table S5: Cognition extraction table.

## Appendix A

### Appendix A.1. Search Terms

The following search terms were entered into the PubMed database searches, * is a wildcard term that searches the database for all variants of the words ending—e.g., for build* the search would look for building, build, builder etc.

Study terms:

- longitudinal study or studies
- prospective study or studies
- retrospective study or studies
- ecological study or studies
- cohort study or studies
- case study or studies
- cross-sectional or cross-sectional study or studies

Noise terms:

- noise
- motorcycle or motorcycles and noise
- environment or environmental noise
- residence characteristics or community noise
- traffic noise
- road noise
- motor vehicle noise
- aircraft noise
- airport noise
- railway noise
- industry noise or industrial noise
- build * noise
- vent * noise
- mechanic * and service noise
- air and condition * noise
- neighbor */neighbor * noise
- train noise
- transportation noise
- leisure activities/leisure time and noise
- low frequency noise
- classroom or school noise
- combined noise
- nuisance noise
- air pollution and noise
- household noise
- wind turbine noise/wind farm noise

Dementia terms:

- dementia
- vascular dementia
- Alzheimer’s disease or Alzheimer disease
- Lewy bodies dementia
- frontotemporal dementia

Cancer terms:

- cancer
- neoplasm
- carcinoma
- sarcoma
- myeloma
- leukemia
- lymphoma

Birth outcomes:

- birth weight
- pregnancy
- fetus/foetus
- preterm
- gestation
- infertility
- sterile
- malformation
- birth
- labor/labour
- prenatal
- perinatal
- fert * or infert *

Mental health, wellbeing, and quality of life:

- mental health
- emotions or emotional disease/disorder
- psychological diagnosis or symptoms
- mental disorders
- psychiatric disorders
- conduct disorder
- anxiety
- depressive disorder or depression
- health status
- wellbeing or well being or well-being
- personal satisfaction
- quality of life
- behavioural or behavioural issues
- helplessness
- strengths and difficulties questionnaire
- kindl
- hrqol
- whoqol
- General health questionnaire or GHQ
- health surveys
- Short Form-36 or SF-36

Cognition:

- executive function
- working memory
- reasoning
- task flexibility *
- problem solv *
- hyperactive *
- concentr *
- speech intelligibility *
- impair *
- standardised assess* or standardized assess *
- SATS/Sats
- reading
- reading comprehension
- oral comprehension
- memory
- attention
- learn impair *

Due to time constraints and the breadth of the PubMed database searches, the Science Direct searches used a sub-set of these search terms to try and identify papers that had not been already identified. The Science Direct searches focused on aircraft noise, road traffic noise, railway noise, and wind-turbine noise for each health outcome.

### Appendix A.2. Table A1

**Table A1:** Excluded papers.

Outcome	Reason for Exclusions
Mental Health	
1. Dzhambov, A., Hartig, T., Markevych, I., Tilov, B., & Dimitrova, D. (2018). Urban residential greenspace and mental health in youth: Different approaches to testing multiple pathways yield different conclusions. *Environmental Research, 160*, 47-59.	Does not report association between noise exposure and mental health
2. Gascon, M., Sanchez-Benavides, G., Dadvand, P., Martinez, D., Gramunt, N., Gotsens, X.,… Nieuwenhuijsen, M. (2018). Long-term exposure to residential green and blue spaces and anxiety and depression in adults: A cross-sectional study. *Environmental Research, 162*, 231-239.	Does not report association between noise exposure and mental health
3. Xiao, J., Li, X., & Zhang, Z. (2016). DALY-Based Health Risk Assessment of Construction Noise in Beijing, China. International Journal of Environmental Research and Public Health, 13(11), doi:10.3390/ijerph13111045	Does not directly measure noise
4. Taskaya, S. (2018). Environmental quality and well-being level in Turkey. Environmental Science and Pollution Research International, 25(28), 27935-27944, doi:10.1007/s11356-018-2806-4	Does not directly measure noise
5. Ma, J., Li, C., Kwan, M. P., & Chai, Y. (2018). A Multilevel Analysis of Perceived Noise Pollution, Geographic Contexts and Mental Health in Beijing. International Journal of Environmental Research and Public Health, 15(7), doi:10.3390/ijerph15071479	Does not directly measure noise
6. Kamimura, A., Armenta, B., Nourian, M., Assasnik, N., Nourian, K., & Chernenko, A. (2017). Perceived Environmental Pollution and Its Impact on Health in China, Japan, and South Korea. Journal of Preventive Medicine and Public Health. Yebang Uihakhoe Chi, 50(3), 188-194, doi:10.3961/jpmph.17.044	Does not directly measure noise
7. Hammersen, F., Niemann, H., & Hoebel, J. (2016). Environmental Noise Annoyance and Mental Health in Adults: Findings from the Cross-Sectional German Health Update (GEDA) Study 2012. International Journal of Environmental Research and Public Health, 13(10), doi:10.3390/ijerph13100954	Does not directly measure noise
8. Dreger, S., Meyer, N., Fromme, H., & Bolte, G. (2015). Environmental noise and incident mental health problems: a prospective cohort study among school children in Germany. Environmental Research, 143, 49-54.	Does not directly measure noise
9. Pun, V. C., Manjourides, J., & Suh, H. H. (2019). Close proximity to roadway and urbanicity associated with mental ill-health in older adults. Science of the Total Environment, 658, 854-860, doi:10.1016/j.scitotenv.2018.12.221	Does not directly measure noise
10. Skrzypek, M., Kowalska, M., Czech, E. M., Niewiadomska, E., & Zejda, J. E. (2017). Impact of road traffic noise on sleep disturbances and attention disorders amongst school children living in Upper Silesian Industrial Zone, Poland. International Journal of Occupational Medicine and Environmental Health, 30(3), 511-520, doi:10.13075/ijomeh.1896.00823	Does not directly measure noise
**Dementia and other neurodegenerative outcomes **	
1. Chen, H., Kwong, J. C., Copes, R., Hystad, P., van Donkelaar, A., Tu, K.,… Burnett, R. T. (2017). Exposure to ambient air pollution and the incidence of dementia: A population-based cohort study. *Environment International, 108*, 271-277.	Does not report association between noise exposure and mental health
2. Chen, H., Kwong, J. C., Copes, R., Tu, K., Villeneuve, P. J., van Donkelaar, A.,… Burnett, R. T. (2017). Living near major roads and the incidence of dementia, Parkinson’s disease, and multiple sclerosis: a population-based cohort study. *Lancet, 389*(10070), 718-726.	Does not measure noise exposure: measures distance to road
**Birth and fertility outcomes**	
1. Robinson, O., Tamayo, I., de Castro, M., Valentin, A., Giorgis-Allemand, L., Hjertager Krog, N.,… Basagana, X. (2018). The Urban Exposome during Pregnancy and Its Socioeconomic Determinants. *Environmental Health Perspectives, 126*(7), 077005.	Does not report a relevant health outcome
2. He, S., Smargiassi, A., Low, N., Bilodeau-Bertrand, M., Ayoub, A., & Auger, N. (2019). Residential noise exposure and the longitudinal risk of hospitalization for depression after pregnancy: Postpartum and beyond. *Environmental Research, 170*, 26-32.	Does not report a relevant health outcome: moved to the mental health review.
3. Nassan, F. L., Chavarro, J. E., Minguez-Alarcon, L., Williams, P. L., Tanrikut, C., Ford, J. B.,… Gaskins, A. J. (2018). Residential distance to major roadways and semen quality, sperm DNA integrity, chromosomal disomy, and serum reproductive hormones among men attending a fertility clinic. International Journal of Hygiene and Environmental Health, 221(5), 830-837.	Does not measure noise exposure: measures distance to road
**Cancer**	
1. Hvidtfeldt, U. A., Sorensen, M., Geels, C., Ketzel, M., Khan, J., Tjonneland, A.,… Raaschou-Nielsen, O. (2019). Long-term residential exposure to PM2.5, PM10, black carbon, NO2, and ozone and mortality in a Danish cohort. *Environment International, 123*, 265-272.	Does not report a relevant health outcome
2. James, P., Hart, J. E., Banay, R. F., & Laden, F. (2016). Exposure to Greenness and Mortality in a Nationwide Prospective Cohort Study of Women. *Environmental Health Perspectives, 124*(9), 1344-1352.	Does not report noise exposure
3. Roswall, N., Andersen, Z. J., von Euler-Chelpin, M., Vejborg, I., Lynge, E., Jensen, S. S.,… Sorensen, M. (2018). Residential traffic noise and mammographic breast density in the Diet, Cancer, and Health cohort. Cancer Causes and Control, 29(4-5), 399-404, doi:10.1007/s10552-018-1021-4	Does not report a relevant health outcome—reports a risk factor for breast cancer not cancer per se.
**Cognition**	
1. Van Aart, C. J. C., Michels, N., Sioen, I., De Decker, A., Bijnens, E. M., Janssen, B. G.,… Nawrot, T. S. (2018). Residential landscape as a predictor of psychosocial stress in the life course from childhood to adolescence. *Environment International, 120*, 456-463.	Does not report a relevant cognitive outcome. Does report mental health but was already identified in the mental health review.
2. Braat-Eggen, P. E., van Heijst, A., Hornikx, M., & Kohlrausch, A. (2017). Noise disturbance in open-plan study environments: a field study on noise sources, student tasks and room acoustic parameters. *Ergonomics, 60*(9), 1297-1314.	Experimental study
3. Connolly, D., Dockrell, J., Shield, B., Conetta, R., Mydlarz, C., & Cox, T. (2019). The effects of classroom noise on the reading comprehension of adolescents. *Journal of the Acoustical Society of America, 145*(1), 372.	Experimental study
4. Forns, J., Dadvand, P., Foraster, M., Alvarez-Pedrerol, M., Rivas, I., López-Vicente, M.,… Sunyer, J. (2016). Traffic-related air pollution, noise at school and behavioural problems in Barcelona schoolchildren: a cross-sectional study. *Environmental Health Perspectives, 124*(4), 529-535	Does not report a relevant cognitive outcome. Moved to mental health review.
5. Silva, L. T., Oliveria, I. S., & Silva, J. F. (2016). The impact of urban noise on primary schools. Perceptive evaluation and objective assessment. Applied Acoustics, 106, 2-9.	Does not report a relevant cognitive outcome. Reports attitudes to noise.
6. Onchang, R., & Hawker, D. W. (2018). Community noise exposure and annoyance, activity interference, and academic achievement among university students. Noise Health, 20(94), 69-76.	Does not report on noise exposure and grade point average (reports on the association for noise annoyance)
