# Supplementary material for: Evidence for Environmental Noise Effects on Health for the United Kingdom Policy Context: A Systematic Review of the Effects of Environmental Noise on Mental Health, Wellbeing, Quality of Life, Cancer, Dementia, Birth, Reproductive Outcomes, and Cognition
Source: Int J Environ Res Public Health. 2020 Jan 7;17(2):393. doi: 10.3390/ijerph17020393 (PMC7013411; doi:10.3390/ijerph17020393)
Supplement: Supplementary file 1 [file ijerph-17-00393-s001.pdf]

International Journal of
Environmental Research and Public Health

MDPI

Evidence for environmental noise effects on health for the United Kingdom policy context: a systematic review of the effects of environmental noise on mental health, wellbeing, and quality of life; cancer; dementia; birth and reproductive outcomes; and cognition (Supplementary Material)

Charlotte Clark* [1], Clare Crumpler [1] and Hilary Notley [2],

[1] Acoustics, Ove Arup & Partners, 13 Fitzroy Street, London, W1T 4BQ, United Kingdom. Charlotte.Clark@arup.com
[2] UK Department for the Environment, Food and Rural Affairs (Defra), www.gov.uk/defra. noise@defra.gov.uk
* Correspondence: Charlotte.Clark@arup.com; Tel.: +44-2077554702.

Supplementary Material

Table S1: Mental health, wellbeing and quality of life extraction table

Reference	Study Design	Population	Exposure	Comparator	Confounding	Outcome	Findings
Weyde, Envt Health, 2017	Cohort study	Based on the Norwegian Mother and Child Cohort Study. Pregnancy sample:	Road and rail traffic noise exposure was modelled using the Nordic Prediction Method	Less than or equal to Lden 30dB	Household income, urbanity, maternal education, ethnicity, maternal alcohol consumption and smoking during pregnancy,	Inattention in 8-year old as reported by mothers	An association with inattention at age 8 years was found for road traffic noise exposure at age 8 years (coef = .0083, CI = [.0012, .0154];

Feder et al., Environ Res, 2015	Cross sectional	Randomly selected participants aged 18–79 (606 males, 632 females)	Outdoor wind turbine sound pressure levels were estimated at each dwelling using both ISO9613-1and ISO9613-2(ISO 1993,1996) as incorporated in the commercial software Cadna Aversion	4 dB	Provincial differences	Quality of life, assessed using the WHOQOL-BREF	Wind turbine noise levels were not found to be related to scores on the Physical, Psychological, Social or Environment domains, or to rated QOL and Satisfaction with Health questions
Seidler et al., Environ Res, 2017	Case control	Individuals aged ≥40 years living in the region of Frankfurt International airport	Address-specific exposure to aircraft, road and railway traffic noise in 2005 was estimated	<40 dB	Age, sex, urban living environment and the local proportion of people receiving unemployment benefit as an indicator of socio-economic status	Diagnoses of depression	For road traffic noise, a linear exposure-risk relationship was found for 24-h continuous sound levels ≥70 dB. For aircraft noise, the risk estimates reached a maximum at 50–55 dB and decreased at higher exposure categories. For railway noise, risk estimates peaked at 60–65 dB
Welch et al., Noise Health, 2018	Case control	Residents aged >18 of Wellington city, New Zealand	Residents living within 250 meters of Wellington airport and within 65 Db Ldn contour (Airport Group)	65 dB	Sex, age, education, employment status, current illness, noise sensitivity	Noise sensitivity – measured using a self-rated three-point scale	People were found to have a significantly poorer Health related QOL than others when they lived near an airport, but not when they lived in the control area

Klatte et al., Environ & Behavior, 2016	Secondary analysis of the NORAH dataset	1,243 second graders from 29 schools around Frankfurt/Main Airport in Germany	Aircraft noise levels were calculated on the basis of radar data from the Flight Track and Aircraft Noise Monitoring System provided by German Air Traffic Services. Road traffic and railway noise levels were estimated using a combination of information provided by local authorities	39 dB	Age, gender, non-verbal abilities, SES, migration background, number of children's books at home, German language proficiency, percentage of children with a migration background in the class, mean SES, class size, and parental involvement, classroom insulation, road-traffic noise, and railway noise at school	Parent ratings of children's quality of life and children's wellbeing in school	Increasing exposure was linearly associated with less positive ratings of quality of life, increasing noise annoyance, and decreasing reading performance. A 20 dB increase in aircraft noise exposure was associated with a decrease in reading scores of one fifth of a standard deviation, corresponding to a reading delay of about 2 months
Dzhambov et al., Environ Res, 2018a	Cross sectional	720 students aged 18-35 years, residing in Plovdiv	Residential noise exposure (LAeq; day equivalent noise level) was obtained by applying a land use regression (LUR) model	62.4 - 73.5 dB	Sex, age, ethnicity, duration of residence, time spent at home/day, and stressful life events	Mental health measured using General Health Questionnaire (GHQ-12)	Evidence that increased residential noise was related to mental ill-health through several indirect pathways
Generaal et al., Psychol Med, 2019	Cross sectional	2980 participants with and without depressive and anxiety disorders in the past year	Daily mean noise of road- rail- and air traffic for several years were modelled by the Netherlands Environmental	N.A	Age, sex, years of education and household income	The presence of current diagnoses of depressive disorders and anxiety disorders	Neighbourhood socioeconomic factors, physical factors (high levels of traffic noise) and social factors (lower social cohesion and less safety) were associated with the

			Assessment Agency by using the Empara Noise tool				presence of depressive and anxiety disorders
Dzhambov et al., Environ Res, 2018b	Cross-sectional	720 students aged 18-35 years, residing in Plovdiv	Residential noise exposure (LAeq; day equivalent noise level) was obtained by applying a land use regression (LUR) model	N.A	Age, sex, ethnicity, individual level, economic status, duration of residence, time spent at home/day, population, and month of data collection	Mental health measured using General Health Questionnaire (GHQ-12)	Evidence that having more greenspace near the residence supported mental health through several indirect pathways with serial components
Dzhambov et al., Environ Int., 2017	Cross sectional	399 students aged 15–25 years, recruited from two high schools and three universities in Plovdiv	Road traffic noise exposure (Lden) was derived from the strategic noise map of Plovdiv	50 dB	Sex, age, ethnicity, socioeconomic status and noise sensitivity	Mental health measured using General Health Questionnaire (GHQ-12)	higher noise exposure was associated with worse mental health only indirectly
Zock et al., Environ Int., 2018	Cross sectional	4450 registered patients of Dutch GPs who were living in 2013 in one of the 181 five-digit postal code areas in the Netherlands	Exposure to road traffic noise and railway noise was estimated by applying the Standard Model Instrumentation for Noise Assessments	N.A	Sex, age, household income, and socio-economic status and municipality and neighbourhood	Diagnosed (co)morbidity and registered symptoms - coded following the International Classification of Primary Care	A high diversity in land use of neighbourhoods may be beneficial for physical and mental health of the inhabitants
Lim et al., Noise Health, 2018	Population-based study	918 elementary and middle-school students in South Korea	The level of road traffic noise at the exterior wall of a residential building	N.A	Age, sex, income, premature birth, maternal age at birth, maternal disease during	Mental health in childhood	Noise sensitivity was significantly associated with internalizing,

			was calculated using noise prediction software based on a noise map		pregnancy, passive smoking, mental disorders		externalizing, and total behavioural problems
Onchang et al., Noise Health, 2018	Cross sectional	Student group residing off campus (n= 450) and a control group residing in dormitories on-campus (n=336)	Noise levels at both on-campus and off-campus locations were measured using sound level meters	N. A	No adjustments made	GPA score	Various contemporary community noise sources affect university students' activities and possibly influence their educational achievement as well
Forns et al., Enviro Health Perspectives, 2016	Cross sectional	Children aged 7-11 years in Barcelona during 2012-13	Noise levels inside the classroom were measured during the second 1-week air pollution sampling period	N. A	Child's sex, child's age, black carbon concentrations at home, traffic noise annoyance at home, home tobacco use, indicators of SES at the individual level and the area level	Total ADHD symptomatology	Noise was significantly associated with ADHD-DSM-IV scores
He et al., Environ Res., 2019	Cohort study	140,456 pregnant women with no documented history of mental illness, who residing in Montreal	Three indicators of noise exposure were used including A-weighted total outdoor noise (LAeq. 24 h), day-evening-night equivalent noise (Lden), and nighttime noise (Lnight)	50 dB	Maternal age, parity, multiple pregnancy, stillbirth, comorbidity, socioeconomic deprivation, neighbourhood walkability and time period	Hospitalizations for depression or other mental disorders	Compared with 50 dB(A), an LAeq. 24 h of 60 dB(A) was associated with 1.16 times (95% CI 0.84–1.62) the risk of depression hospitalization, and 1.34 times (95% CI 1.04–1.74) the risk of other mental disorders

Civil Aviation Authority, 2017 (Survey of Noise Attitudes)	Cross-sectional study	1877 adult participants living near Birmingham; East Midlands; Gatwick; Heathrow; London City; Luton; Manchester; Newcastle; Stansted airports	Respondents were selected based on exposure of 51dB LAeq16 hour (92-day average) or higher for summer 2013 using published noise contour data for the airport	Sampling was stratified so that one-third of the sample was exposed to 51-54dB LAeq16 hour, and two-thirds were exposed to >54dB LAeq16 hour	None	Warwick Edinburgh Mental Wellbeing Scale and self-reported health	Found no association between aircraft noise (LAeq 16h 92 day) and wellbeing or self-reported health
Klompmaker et al., 2019, Environment International	Cross sectional	National public health survey in Netherlands which includes information on 387,195 citizens, aged ≥19 years	Residential traffic noise levels were estimated by the Standard Model Instrumentation for Noise Assessments	N.A	Age, sex, marital status, region of origin, paid occupation, household income, level of education, neighbourhood SES, smoking status, alcohol use, degree of urbanization	Mental health, measured using the Dutch national health survey	Road-traffic noise was only positively associated with prescription of anxiolytics, while rail-traffic noise was only positively associated with psychological distress
Enembe et al., 2018, Environmental International	Cross sectional	Eight-thousand Helsinki residents ag 25 years and above were selected from the Population Registry of Finland	Residential exposure to road-traffic noise was estimated from façade noise maps	<45 dB	Sex, age, marital status, employment status, household income, alcohol intake, current smoking status, level of physical activity, pet ownership and sleep disturbance	Use of sleep medication, anxiolytics and antidepressants	Noise annoyance was associated with anxiolytic drug use, OR=1.41 (95% CI: 1.02–1.95), but not with sedative or antidepressant use. There was suggestive association between modelled noise at levels higher than 60 dB and anxiolytic or antidepressant use

Oiamo et al., 2015, Social Science and Medicine	Cross sectional	603 individuals that were exposed to traffic noise and air pollution in Windsor, Ontario, Canada	Residential levels of traffic noise were modelled in SoundPLAN 7.3	N.A	Sex & age	Health related quality of life measured by the SF-12 health survey	Noise annoyance had a significant and negative effect on both mental and physical health factors of the SF-12 and there was a significant covariance between noise annoyance and odour annoyance
Leijssen et al., 2019, International Journal of Hygience and Environmental Health	Cross sectional	23,293 participants, aged 18-70 years, living in Amsterdam between 2011 and 2015	Modelled daily average noise levels on road-traffic for the year 2011 in the Netherlands using the Empara Noise tool	45 dB	Age, sex, educational level, occupational status, ethnic origin, marital status, household composition, neuroticism, stressful life events, neighbourhood-level, including socioeconomic status, blue/green space and livability	Depressed mood	Exposure to ≥70 dB(A) compared to the reference group of 45–54 dB(A) showed a significant positive association with depressed mood (OR: 1.65, 95% CI 1.10, 2.48)
Zijlema et al., 2015, Int. Journal of Hygiene and Environmental Health	Cross sectional	5,304 participants, aged between 18-92 years, in the Netherlands	Road traffic noise was estimated using a new implementation of the CNOSSOS-EU noise modelling framework	N. A	Sex, age, educational level and household equivalent income, hostility and vulnerability to stress	Somatic symptoms	No association of noise exposure and somatic symptoms (incidence rate ratio (IRR) 1.001; 95% confidence interval (CI) 1.000–1.001; n = 56,937)

Wallas et al., 2018, Int. Journal of Hygiene and Environmental Health	Cross sectional	1751 adolescents from the BAMSE birth cohort based in Stockholm County, between 1994-1996	Traffic noise exposure assessment was performed using data from several national, regional and local authorities	<45 dB	Age, sex, rhinitis, eczema and sampling season	Saliva cortisol levels	Road traffic noise exposure was not associated with saliva cortisol, however, annoyance to noise tended to increase the levels. Saliva cortisol levels appeared particularly high among those who were highly annoyed and exposed to road traffic noise levels≥55 dB Lden
Lawton et al., 2016, Transportation Research Part D	Cross sectional	Two-year sample of nearly 190,000 households, from the Annual Population Survey, UK	Noise contour data were derived from annual average noise levels from 2012 airport operator strategic noise maps at the geographical level of residential dwelling outer area	55 dB	Ethnicity, household income, health status, marital status, employment status, housing status, gender, age, geographic region, religion, and education	Subjective wellbeing	The presence of daytime aviation noise was found to consistently negatively impact on five subjective wellbeing measures
Wright et al. 2018, Environmental Health	Cross sectional	198,532 people enumerated at the 2011 Census, aged 18 years and over and living within the 54 dB Belfast City Airport noise contour	Residential exposure to aircraft noise (LAeq,16h) was assessed by linking Census records with modelled noise contours surrounding George Best Belfast City Airport	54 dB	Age, sex, ethnicity, religion and marital status, education, property value and car availability, likelihood of poor mental	Prevalence of self-assessed mental ill health	No association between aircraft noise and risk of mental ill health

Van Aart et al., Environ Int., 2018	Longitudinal	172 Belgian children aged 6.7-12.2, followed for three years (2012-2015)	A GIS-based noise model including the Flemish street and railway networks was used to estimate traffic noise levels in 5 dB(A)-intervals according to the European Noise Directive (2002/49/EC)	N.A	Age, sex & socioeconomic status	Childhood psychosocial stress – Strengths and Difficulties questionnaire and hair cortisol	Inverse association between residential and traffic density with hyperactivity problems

Table S2: Cancer extraction table

Reference	Study Design	Population	Exposure	Comparator	Confounding	Outcome	Findings
Andersen et al., Lynge Breast Cancer Res, 2018	Longitudinal cohort study	22,466 female nurses (age > 44 years) who at recruitment in 1993 or 1999 reported information on breast cancer risk factors	Road traffic noise levels at the nurse road traffic noise levels at the nurses' residential addresses were calculated using the Nord2000	<48 dB	Age, birth cohort, body mass index, alcohol use, leisure time physical activity, smoking status, age at menarche, parity (yes; no), number of children, age at first birth, menopausal status, HT use, and oral contraceptive use	Incidence of breast cancer	For each 10 dB increase in 24-year mean noise levels at their residence, a statistically significant 10% increase in total breast cancer incidence was found
Hegewald et al., Scandinavian J Work Envt Health, 2017	Prospective	1,026 ,670 Women aged ≥40 years by 2010 living in the region surrounding the Frankfurt international airport	Aircraft noises, road traffic noise and rail noise were all modelled using SoundPLan.	<40 dB	Age, hormone replacement therapy, and regional proportion of people receiving unemployment benefits	Incident diagnoses of breast cancer	An increased odds ratio was observed for estrogen receptor negative tumors at 24-hour aircraft noise levels 55–59 dB [OR 55–59 dB 1.41. Clear associations between road and rail traffic noise were not observed

Roswall et al., Environ Reseasrch, 2016	Longitudinal cohort study	57,053 participants (29,875 women) aged 50-64 years of age residing in Copenhagen	Residential road traffic noise was calculated as the equivalent continuous A-weights sound pressure level (LAeq)	N. A	Calendar year at diagnosis, train noise, smoking, alcohol intake, abstainers, waist circumference, recreational physical activity and marital status	Overall mortality and breast cancer-specific mortality	No association was found between time-weighted averages of residential road traffic noise 1-,3- or 5- years before death and overall or breast cancer-specific mortality
Roswall, et al., Cancer, Causes & Control, 2017	Longitudinal cohort study	57,053 participants (29,875 women) aged 50-64 years of age residing in Copenhagen	Traffic noise was calculated for all residential addresses from 1987 to 2012 for 51,283 Danes in the Diet, Cancer and Health Cohort. Railway noise also calculated	<40 dB	Age, sex, railway noise, smoking, smoking duration, smoking intensity, alcohol intake, abstainers. recreational physical activity, education, whole grain cereal, red meat, and marital status, income and municipal-level population density at baseline	Overall colorectal cancer incidence	No association found between residential road traffic noise and rectal cancer. Observed an association with distal colon cancer, but not for proximal colon cancer: 0.99 (0.83–1.18), per 10 dB, 10 years preceding diagnosis. There was no association between railway noise and colorectal cancer, or any subtype

Roswall et al., PloS One, 2015	Longitudinal cohort study	27,178 men aged 50-65 years born in Denmark with no previous cancer diagnosis and living in Greater Copenhagen	Road and railway traffic was calculated using SoundPLAN	N. A	Education level, area level socioeconomic position of baseline municipalities or districts for Copenhagen municipality based on municipality/district information on education; work market affiliation; income; smoking status; smoking duration; body mass index; waist circumference; physical activity; calendar year; and airport noise	Incidence rate ratios for association between road traffic and railway noise and prostate cancer	There was no association between residential road traffic noise and risk of prostate cancer for any of the three exposure windows. For railway noise, there was no association with overall prostate cancer
Roswall et al., PloS One, 2017	Longitudinal cohort study	57,053 participants (29,875 women) aged 50-64 years of age residing in Copenhagen	Road and railway traffic was calculated using SoundPLAN	N. A	Age, calendar year of diagnosis and sex, railway noise at diagnosis, baseline smoking status, baseline smoking duration, baseline alcohol intake, baseline abstainers, baseline red meat intake, baseline recreational physical activity, education 1 year before diagnosis and income 1 year before diagnosis	Overall mortality and colorectal cancer-specific mortality	No association was found between road traffic noise and overall (MRR 1.00 (0.88-1.13) per 10dB) or colorectal cancer – specific mortality (MRR 0.98 (0.85-1.13) per 10 dB) over the entire follow-up period, or 1 years preceding death. Railway noise was only included as a covariate

Sorensen et al., I J of Cancer, 2014	Longitudinal cohort study	(29,875 women) aged 50-64 years of age residing in Copenhagen	Road traffic and railway traffic noise exposure were calculated using SoundPLAN	<42 dB	Age, parity, age at first birth, hormone replacement therapy status and duration, age at menarche, length of school attendance, BMI, alcohol consumption, alcohol intake, smoking status, intake of vegetables, physical activity, calendar-year and railways and airport noise	Incidence rate ratios (IRRs) for breast cancer in association with road traffic and railway noise	No overall association was found between residential road traffic or railway noise and breast cancer risk. Among women with estrogen receptor negative breast cancer, a 10-dB higher level of road traffic noise during the previous 1, 5 and 10 years were associated with 28%, 23% and 20% higher risks of estrogen receptor negative breast cancer. Similarly, a 10-dB increase in railway noise increased risk for estrogen receptor negative breast cancer by 38%. No association was found between road traffic or railway noise and estrogen receptor positive breast cancer

Sorensen et al., Environmental Research, 2015	Case control	2753 Cases were identified using the Cancer Registry. Eligible cases were Danes between 30 and 84 years of age with a primary diagnosis of NHL between 1992 and 2010. For each case, two random controls, matched on sex and year of birth were selected from the Civil Registration System	Road traffic noise exposure was calculated at the most exposed façade for all present and historical addresses using Sound PLAN	55 dB	Age and sex, education, disposable income, cohabitation status, Charlson comorbidity index, air pollution	Odds ratios and 95% confidence internals for risk for non-hodgkin lymphoma associated with exposure to traffic noise	A 5—year time-weighted mean of road traffic noise about 65dB was associated with an 18% higher risk for non-hodgkin lymphoma (NHL) when compared to road traffic noise below 55dB, whereas for exposure between 55 and 65dB no association was found. In analyses of NHL subtypes, no association was found between road traffic noise and risk of T-cell lymphoma, whereas increased risks for B-cell lymphoma and unspecified lymphomas were observed as exposures above 65dB

Table S3: Dementia and other neurodegenerative outcomes extraction table

Reference	Study Design	Population	Exposure	Comparator	Confounding	Outcome	Findings
Andersson et al., Environmental Research, 2018	Longitudinal cohort study	1721 participants from the Betula project, 985 men and 736 women, who at baseline were aged 55–85 years (mean 68.5 ± 9.4)	Modelled data provided road traffic noise levels (Leq. 24 h) at the participants' residential address at baseline	35 dB	Age, ApoE4, education, physical activity, smoking, sex, alcohol use, BMI, and waist-hip ratio, hypertension, diabetes, and stroke	Dementia incidence	Exposure to noise levels (Leq. 24 h)> 55 dB had no significant effect on dementia risk (HR 0.95; CI: 0.57, 1.57).
Carey et al., BMJ Open, 2018	Retrospective cohort study	130 978 adults aged 50–79 years registered with their general practices on 1 January 2005, with no recorded history of dementia or care home residence	Traffic intensity, distance from major road and night-time noise levels (Lnight) were estimated at the postcode level linked to clinical data via residential postcode. Road traffic noise levels were estimated using the TRAffic Noise EXposure (TRANEX) model	Mean exposure Lnight 52.1dB	Age, sex, ethnicity, smoking and body mass index, area deprivation and comorbidity (IHD, stroke, diabetes, heart failure)	A first recorded diagnosis of dementia and, where specified, subgroups of Alzheimer's disease and vascular dementia during 2005–2013	There was a positive exposure response relationship between dementia and all measures of air pollution except O3. Increases in dementia risk were also observed with PM2.5, PM2.5 specifically from primary traffic sources only and Lnight, but only NO2 and PM2.5

Culqui et al., Science of Total Environment, 2017	Longitudinal ecological time series study	Madrid during the period 2001-2009 – mean population of 3,116,897 and of this total, 754,005 persons (24.2%) were aged 60 years or over	Mean daily noise levels (dB(A)) for equivalent diurnal noise level 7–23 h (Leqd), equivalent nocturnal noise level 23–7 h (Leqn), and daily noise level 24 h (Leq24) were measured	N. A	No adjustments made	Short-term admissions to hospital for Alzheimer's disease (ICD-9 code)	There was no statistically significant association with emergency Alzheimer's disease admissions or noise
Linares et al., Environ Res.,2017	Longitudinl ecological time-series study	Number of daily dementia-related emergency (DDE) hospital admissions to Madrid municipal as obtained from the Hospital Morbidity Survey (National Statistics Institute)	Leqd, equivalent diurnal noise level (from 8 to 22 h), and Leqn, equivalent nocturnal noise level (from 22 to 8 h) in dB(A) was provided by the Madrid Municipal Air Quality Monitoring Grid	N. A		Number of daily dementia-related emergency admissions to municipal hospitals in Madrid	Admissions displayed a linear functional relationship without a threshold in the case of Leqd. The RR of DDE admissions was 1.15 (1.11–1.20) for an increase of 1 dB in Leqd

Diaz J et al., 2017 Gac Sanit	Ecological time series analysis	The population of Madrid during the period 2001-2009, it had a mean population of 3,116,897 and of this total, 284,929 persons (9%) were aged 75 years or over	The Madrid Municipal Air Quality Monitoring Grid supplied Leqd, equivalent diurnal noise level (from 8 to 22 h), and Leqn, equivalent nocturnal noise level	55 dB	Temperature, pollution, trends and seasons	Parkinson's Disease related demand for healthcare	The association between Leqd and Hospital admissions was found to be linear. Leqd and Leqn at lag 0.1 and temperature at lags 1 and 5 were the only environmental variables associated with increased Parkinson's disease related healthcare demand
Carmona et al., 2018 Science of the Total Environment	Longitudinal ecological time series study	The population of Madrid during the period 2001-2009, it had a mean population of 3,116,897	Mean daily noise levels (dB(A)) for equivalent diurnal noise level 7–23 h (Leqd), equivalent nocturnal noise level 23–7 h (Leqn), and daily noise level 24 h (Leq24), supplied by the Madrid Municipal Air Quality Monitoring Grid	N. A	Linear trends, seasonality and the autoregressive nature of the series itself	Number of emergency MS hospital admissions	Traffic noise can exacerbate MS symptoms, leading to hospital admissions due to this cause

Tzivian et al., Environmental Health Perspectives, 2016	Cross-sectional cohort study	4,086 participants who were 50–80 years old	Lden & Lnight – long term exposure at the baseline address (2000-2003)	Lden mean 53.74 (SD+-9.49)<br>Lnight mean 44.88 (SD+-9.17)	Age, sex, socioeconomic status, alcohol consumption, smoking status, self-reported environmental tobacco smoke, any regular physical activity, body mass index	Diagnoses of Mild cognitive impairment (MCI)	A 10 dB(A) increase in LDEN was associated with overall MCI. A 10dB(A) increase in Lnight was associated with overall MCI

61 **Table S4: Birth and reproductive outcomes extraction table**

Reference	Study Design	Population	Exposure	Comparator	Confounding	Outcome	Findings
Hjortebjerg et al., Scand J Work Environ Health, 2018	Cohort study	Study based on children enrolled in the Danish National Birth Cohort which consecutively recruited pregnant women from March 1996 to November 2002 from all over Denmark. 57,282 children participated	Estimated annual levels of road traffic and railway noise at all addresses using SoundPLAN	N. A	Age, sex of child, maternal age at birth, parity, smoking during first trimester, alcohol consumption during first trimester, level of education, disposable income one year before birth of the child, road traffic noise for air pollution and vice versa	Children with febrile seizures were identified by linking the unique personal identification number of each child in the study base to the nationwide Danish National Patient Register	An interquartile range increase in childhood exposure to road traffic noise and air pollution was associated with an 11% and 5% higher risk for febrile seizures, respectively, after adjustment for potential confounders

Min KB & Min JY, Environ Pollut, 2017	Population based cohort study	Used the National Health Insurance Service-National Sample Cohort (2002-2013), a population-wide health insurance claims dataset. A total of 206,492 males of reproductive age (20-59 years) with no history of congenital malformations	Data on noise exposure was obtained from the National Noise Information System	35 dB	Age, income, residence area, smoking history, exercise, alcohol use, blood glucose levels, BMI, history of diseases	Diagnoses of male infertility	A non-linear dose-response relationship was observed between infertility and quartiles of daytime and night time noise after adjustment for confounding variables. Based on WHO criteria, adjusted odds for infertility were significantly increased (OR ¼ 1.14; 95% CI, 1.05e1.23) in males exposed to night time [illegible]
Pedersen et al., Environ Res., 2017	Cohort study	84,218 liveborn singletons (1997–2002) from the Danish National Birth Cohort	Road traffic noise was calculated at the most exposed facade of each residential address using SoundPLAN	N. A	Maternal smoking, maternal alcohol consumption, parental age (years), maternal education the year before last menstrual period, household disposable income, parity, maternal pre-pregnancy BMI	Diagnoses of congenital anomalies	Residential road traffic exposure to noise or air pollution during pregnancy did not increase risk for development of congenital anomalies

Smith et al., BMJ, 2017	Retrospective population-based cohort study	540,365 singleton term live births	A-weighted road traffic noise levels (dB) were modelled to 0.1 dB resolution for all geocoded maternal residential addresses using the Traffic Noise Exposure (TRANEX) model	50 dB	Maternal age, birth registration type, birth season, birth year, Carstairs deprivation quintile, tobacco expenditure. Birth weight and LBW were adjusted for sex, gestational age and baby's ethnicity	Birth weight outcomes – low birth weight, small for gestational age	Trends of decreasing birth weight across increasing road traffic noise categories were observed, but were strongly attenuated when adjusted for primary traffic related air pollutants
Dzhambov et al., 2019, Science of the Total Environment	Explorative study	Used data from two cross-sectional studies (UIT, n = 573 and BBT, n = 518) in the Tyrol Region (Austria/Italy)	Noise emissions were calculated in 2003/2004.Total day-evening night noise level (Lden) was calculated at the most exposed façade covering road and rail noise	10 dB	sex of child, age of mother at birth, gestational age, single mother status, mother's education, smoking during pregnancy, duration of residence before conception, and house type, Ldn or NO2	Birth outcomes – low birth weight and small for gestational age	An increase of Ldn was associated with higher odds for low birth weight but only in one of the studies. Unexpectedly, an increase in Lden was associated with an increase in the odds for being small for gestational age.

Poulsen et al., 2018, Environmental Research	Nationwide registered based study	135,795 pregnant women living in Danish dwellings from 1982 to 2013	Estimated hourly outdoor and low frequency indoor wind turbine noise at the dwellings of the pregnant women and aggregated as mean night-time Wind turbine noise during pregnancy	>24 dB	Sex, calendar year of birth, maternal age at birth, parity, season of conception, marital status, education, work status, personal income, area-level mean disposable income, ever living on a farm during pregnancy, shortest distance to road with ≥5000 vehicles per day during pregnancy, traffic load within 500m radius of dwelling averaged over all addresses held during pregnancy, maternal smoking during 1st trimester and for both maternal	Small for gestational age, pre-term birth and birth weight	No association between night-time wind turbine noise and adverse birth outcomes
Wallas et al., Environ Res., 2019	Longitudinal cohort study	4089 children born 1994-1996 in four pre-defined areas of Stockholm County	Road traffic noise levels were estimated at the most exposed façade of all residential homes where the study subjects lived. Maternal occupational noise exposure during pregnancy was estimated based on self-reported occupation	N. A	Parental occupation, smoking during pregnancy, maternal BMI and municipality at birth	Data on BMI from birth to adolescence were collected via questionnaires, clinical examinations and health care records. A national register provided information on birth outcomes	Residential road traffic noise exposure was associated with increases in BMI from school age to adolescence, but not at earlier ages. Maternal noise exposure during pregnancy was generally unrelated to adverse birth outcomes (low birth weight, pre-term birth) and BMI from birth to adolescence in the children, however, traffic noise exposure was associated with a decreased risk of preterm birth

Table S5: Cognition extraction table

Reference	Study Design	Population	Exposure	Comparator	Confounding	Outcome	Findings
Papanikolaou et al., Int J Adolesc Med Health, 2015	Cross sectional	676 participants (324 boys,47.9% and 352 girls, 52.1%) of the 4th and 5th elementary classes	Selected schools on the basis of increasing levels of exposure to road traffic noise and classified into three categories, according to external noise: Low-level noise: 55–66 dB, Medium-level noise: 67–77 dB, and High-level noise: 72–80 dB. Noise levels outside classrooms were measured with an echo	55-66 dB	No adjustments made	To assess the effects of noise on cognitive skills, a test was constructed based on the National Curriculum for Elementary Education for reading and mathematics	Children in low-level noise schools showed statistically significant differences from children in medium- and high-level noise schools in reading performance ($p < 0.001$). Children in low-level noise schools differed significantly from children in high-level noise schools but only in mathematics performance ($p = 0.001$)

Tzivian et al., Environ Health Perspectives 2016	Cross sectional	4,814 randomly chosen men and women who were 45–75 years old at baseline, enrolled into the study between December 2000 and August 2003	Long-term exposure to traffic noise was modeled according to the European Directive 2002/49/EC as the weighted 24-hr mean (LDEN) and the night-time mean (LNIGHT) at the baseline address	N. A	Age, sex, socioeconomic status, alcohol consumption, smoking status, self-reported environmental tobacco smoke, any regular physical activity, body mass index, background NO2	Assessment of overall mild cognitive impairment (MCI) and amnestic (aMCI) and nonamnestic (naMCI) mild cognitive impairment	Most air pollutants and traffic noise were associated with overall MCI and aMCI. 10 A-weighted decibel [dB(A)] increase in LDEN was associated with overall MCI
Tzivian et al., J Toxicol Environ Health A, 2017	Cross sectional	4814 randomly chosen men and women aged between 45 and 75 years	Long-term exposure to traffic noise was modeled according to the European Directive 2002/49/EC	N. A	Age, sex, socioeconomic status, alcohol consumption, smoking status, ETS, any regular physical activity, and BMI	Cognitive function – measured through a cognitive performance assessment	High noise exposure increased the association of air pollution with cognitive function. observed stronger negative associations in participants with double exposure compared to the addition of effect estimates of each single exposure

Klatte et al., Environ & Behavior, 2016	Secondary analysis of the NORAH dataset	1,243 second graders from 29 schools around Frankfurt/Main Airport in Germany	Aircraft noise levels were calculated on the basis of radar data from the Flight Track and Aircraft Noise Monitoring System provided by German Air Traffic Services. Road traffic and railway noise levels were estimated using a combination of information provided by local authorities and were used only as covariates	39 dB	Age, gender, non-verbal abilities, SES, migration background, number of children's books at home, German language proficiency, percentage of children with a migration background in the class, mean SES, class size, and parental involvement, classroom insulation, road-traffic noise, and railway noise at school	Reading performance - assessed though a standardised reading comprehension test for primary school children instructed in German	Increasing exposure was linearly associated with less positive ratings of quality of life, increasing noise annoyance, and decreasing reading performance. A 20 dB increase in aircraft noise exposure was associated with a decrease in reading scores of one fifth of a standard deviation, corresponding to a reading delay of about 2 months
Spilski et al., ICBEN, 2017	Cross sectional	1,243 children participated in the study	Exposure levels at schools and at the children's homes were assessed by the NORAH acoustic team. Aircraft noise levels were calculated on the basis of radar data from the Flight Track and Aircraft Noise Monitoring System (FANOMOS), provided by German Air Traffic Services	34 dB	Age, gender, SES, migration background, German language proficiency, number of children's books, non-verbal abilities, story comprehension, phonological awareness, access to phonological representations, class socioeconomic status (SES), class size, percentage of children with a migration background, parental involvement in school affairs, classroom insulation, road-traffic noise, and railroad noise at school	Cognition and quality of life	A 10 dB increase of aircraft noise at school was associated with a decrease in children's global reading and word reading scores by one tenth of a SD - one point on the T-score scale. For text reading, a 10 dB increase of aircraft noise was associated with a decrease by one eighth of a SD. For sentence reading, the effect of aircraft noise did not reach significance, neither in the unadjusted model nor in the adjusted models

Spilski et al., Internoise, 2017	Cross sectional	439 German second-graders from 29 schools in the vicinity of Frankfurt Airport, Germany	Aircraft noise - Noise Exposure levels were calculated on the basis of radar data from the Flight Track and Aircraft Noise Monitoring System (FANOMOS) provided by German Air Traffic Services. Road traffic and railway noise levels were estimated using a combination of information by local authorities	39 dB	Age, gender, SES, number of children's books, non-verbal abilities, story comprehension, phonological awareness, access to phonological representations, class SES, class size, percentage of children with a migration background, parental involvement in school affairs, classroom insulation, road-traffic and railway noise at school	Reading comprehension – measured using the Suffolk Reading Scale	A 1 dB increase in LAeq is associated with an increase in distraction of .147 scale points. Fully-adjusted multilevel models showed that LAmax and Emergence (separately calculated models) are significant predictors of distraction of children due to aircraft noise (mediator); which in turn has a significant impact on reading performance
Seabi J et al., Expo Sci Environ Epidemiol. 2015	Longitudinal field study	Cohort of 732 learners with a mean age of 11.1 years participated at baseline measurements in 2009 and 650 and 178 learners were reassessed after the relocation of the airport in 2010 and 2011, respectively	Aircraft noise - To measure the external noise surrounding the five schools, a SVAN 955 Type 1 sound lever meter was utilised	54.4–55.3 Leq and 73.2–74.3 Lamax	Gender, deprivation, language spoken at home and groups on reading comprehension in 2010 and 2011	Reading comprehension – measured using the Suffolk Reading Scale	Results revealed no significant effect of the groups on reading comprehension across the testing periods, but significant effects of home language were demonstrated on reading comprehension

Silva et al., 2016, Applied Acoustics	Cross sectional	The survey covered nine classes located in three primary schools	A sound-level meter of accuracy class 1 was used to measure noise levels	55 dB	No adjustments made	Impact of noise – measured through subjective and objective evaluation	Measurements of indoor and outdoor noise suggest that noise from the outside (road, schoolyard) affects the background noise level in classrooms but in varying degrees
Eagen et al., 2017	Cross-sectional observational field study	134 1½ hour classroom observation sessions of 2nd, 3rd, 4th and 5th Grade children attending schools near Los Angeles airport (LAX)	11 schools from 2 school districts: one school district subject to noise primarily from arrivals operations at LAX. Other school district not heavily influenced by LAX operations	Short-term LAeq 1s, 5s, 10s, 30s; TA (time above) 55dB, 60dB and 65dB; and NA (number above) 55dB, 60dB and 65dB	School level assessment of free school meals, ethnicity, English Language Learners.	Student distracted or interrupted by a noise source (observed). Also observed teaching voice raising and voice masking behaviour	Short-term exposure to aircraft noise events and teacher voice masking and voice raising behaviour but no effect on student distraction
